# Diffusing Capacity for Carbon Monoxide Predicts Response to Balloon Pulmonary Angioplasty in Patients With Inoperable Chronic Thromboembolic Pulmonary Hypertension

**DOI:** 10.3389/fcvm.2021.762267

**Published:** 2021-12-02

**Authors:** Xin Li, Yi Zhang, Qin Luo, Qing Zhao, Qixian Zeng, Tao Yang, Qi Jin, Lu Yan, Anqi Duan, Jiaran Liu, Chenhong An, Xiuping Ma, Changming Xiong, Zhihui Zhao, Zhihong Liu

**Affiliations:** ^1^Center for Pulmonary Vascular Diseases, National Center for Cardiovascular Diseases, Fuwai Hospital, Chinese Academy of Medical Sciences and Peking Union Medical College, Beijing, China; ^2^Department of Cardiology, Zhongshan Hospital, Fudan University, Shanghai, China

**Keywords:** chronic thromboembolic pulmonary hypertension, diffusing capacity for carbon monoxide, balloon pulmonary angioplasty, right heart catheterization, microvasculopathy

## Abstract

**Background:** The hemodynamic results of balloon pulmonary angioplasty vary among patients with inoperable chronic thromboembolic pulmonary hypertension (CTEPH). Previous studies revealed that microvasculopathy accounted for residual pulmonary hypertension after pulmonary endarterectomy, which could be reflected by the diffusing capacity for carbon monoxide (DLCO). We aimed to identify whether the DLCO could predict the BPA response.

**Materials and Methods:** We retrospectively analyzed 75 consecutive patients with inoperable CTEPH who underwent BPA from May 2018 to January 2021 at Fuwai Hospital. According to the hemodynamics at follow-up after the last BPA, patients were classified as “BPA responders” (defined as a mean pulmonary arterial pressure ≤ 30 mmHg and/or a reduction of pulmonary vascular resistance ≥ 30%) or “BPA nonresponders.”

**Results:** At the baseline, BPA responders had significantly higher DLCO values than nonresponders, although the other variables were comparable. In BPA responders, the DLCO decreased after the first BPA session and then returned to a level similar to the baseline at follow-up. Conversely, the DLCO increased constantly from the baseline to follow-up in nonresponders. Multivariate logistic analysis showed that a baseline DLCO of <70% and a percent change in DLCO between the baseline and the period within 7 days after the first BPA session (ΔDLCO) of > 6% were both independent predictors of an unfavorable response to BPA. Receiver operator characteristic analysis showed that the combination of a baseline DLCO < 70% and ΔDLCO > 6% demonstrated a better area under the curve than either of these two variables used alone.

**Conclusions:** A baseline DLCO < 70% and ΔDLCO > 6% could independently predict unfavorable responses to BPA. Measuring the DLCO dynamically facilitates the identification of patients who might have unsatisfactory hemodynamic results after BPA.

## Introduction

Chronic thromboembolic pulmonary hypertension (CTEPH) is characterized by an organic thromboembolic obstruction of proximal pulmonary arteries and secondary microvasculopathy in nonoccluded areas (1). Although pulmonary endarterectomy (PEA) can effectively alleviate thromboembolic obstruction in proximal pulmonary arteries, it cannot address distal lesions. Moreover, 17–51% of patients still suffer from residual pulmonary hypertension after PEA, which is attributed to advanced microvasculopathy (2). Microvasculopathy compromises the pulmonary membrane diffusion capacity, which could be reflected in a decreased diffusing capacity for carbon monoxide (DLCO) (3, 4).

In patients for which PEA is not appropriate, balloon pulmonary angioplasty (BPA) serves as an alternative treatment and could effectively dilate surgically inaccessible pulmonary arteries (5). Similar to PEA, the hemodynamic results of BPA varied among patients, with some patients showing persistent pulmonary hypertension and exercise intolerance even after several BPA sessions (6). Identifying the predictors of hemodynamic response is essential for guiding the clinical practice of BPA, which has received limited research attention. Akizuki et al. reported that BPA had an immediate effect on the DLCO, and that this effect varied with the lung fields (7). Considering its close relationship with microvasculopathy, we hypothesized that the baseline DLCO and the DLCO percent change between the baseline and the period within 7 days after the first BPA session [ΔDLCO = (DLCO after the first BPA session-DLCO before the first BPA session)/DLCO before the first BPA session] could predict the hemodynamic response to BPA.

## Materials and Methods

### Study Design and Participants

This current retrospective cohort study was performed in Fuwai Hospital, Chinese Academy of Medical Sciences (Beijing, China). The study protocol was approved by the Ethics Committee of Fuwai Hospital (Approval No: 2020-1275). Each patient provided signed informed consent. We screened all patients with CTEPH who underwent BPA from May 2018 to January 2021 at Fuwai Hospital. The diagnosis of CTEPH was consistent with the 2015 European Society of Cardiology/ERS guidelines (8), and eligibility for BPA was determined after a discussion among multidisciplinary specialists, including pulmonary vascular specialists, PEA surgeons, and interventional cardiologists. Patients without DLCO records at the baseline or after the first BPA session and patients without right heart catheterization (RHC) at follow-up were excluded. The pulmonary function test, cardiopulmonary exercise test, 6-min walk distance (6 MWD), World Health Organization functional class (WHO-FC), and echocardiography results were evaluated within a week prior to and after each BPA session. Patients were reevaluated over 3 months after the last BPA session. Clinical data were collected from electronic medical records by two independent reviewers. Any discordance was resolved by the supervisors (ZZ and ZL).

### RHC and BPA Procedure

RHC and BPA were performed as previously described (9). RHC was performed prior to each BPA session to obtain the hemodynamics parameters, including the right atrial pressure, right ventricular pressure, mean pulmonary arterial pressure (mPAP), pulmonary arterial wedge pressure, cardiac output (calculated by Fick's method), and oxygen saturation. Mixed venous oxygen saturation (S_v_O_2_) and pulmonary vascular resistance (PVR) were calculated in accordance with standard equations (10). A six Fr guiding catheter [Multipurpose (Cordis Corporation, Bridgewater, New Jersey, USA), Amplatz left catheter (Terumo® Heartrail™ II; Terumo Corporation, Tokyo, Japan) or Judkins right catheter (Terumo® Heartrail™ II; Terumo Corporation, Tokyo, Japan)] was introduced to the pulmonary artery through a seven Fr long sheath (Flexor® Check-Flo® Introducer; Cook Medical, Bloomington, IN, USA). The intervention target was determined based on both pulmonary angiography and lung ventilation/perfusion scintigraphy. Then, a 0.014-inch guide wire (Hi-Torque Pilot 50; Abbot, Santa Clara, CA, USA) was introduced across the target lesion, and, subsequently, the balloon was inflated to dilate the target vessel. A 2 mm × 20 mm balloon was initially used to reduce the risk of complications. In repeated dilations, the inflation pressure and balloon size were dynamically adjusted according to the vessel size and vascular response. For patients with mPAP <30 mmHg, we will increase balloon size up to the reference vessel diameter. For patients with mPAP between 30 and 40 mmHg, we will increase balloon size up to 80% of the reference vessel diameter. For patients with mPAP over 40 mmHg, we prefer to use a small balloon to avoid complications. After each BPA session, the hemodynamics parameters were measured again by RHC.

### Pulmonary Function Tests and Cardiopulmonary Exercise Test

Pulmonary function tests and exercise tests were performed using the COSMED Quark CPET system (COSMED, Rome, Italy) as described previously (11). Forced vital capacity, forced expiratory volume in 1 s, and single-breath DLCO were measured according to the American Thoracic Society/European Respiratory Society criteria (12, 13). To eliminate individual differences, the DLCO reported in the present study was the percentage of predicted DLCO. Peak oxygen consumption (VO_2_@Peak) was defined as the highest 30-s average oxygen consumption after reaching the anaerobic threshold. Both the V-slope method and ventilator equivalents were applied to detect anaerobic thresholds. Ventilatory efficiency was measured by the minute ventilation to the carbon dioxide output (VE/VCO_2_) ratio and represented by the VE/VCO_2_ slope during the incremental exercise.

### Definition of BPA Responders and Nonresponders

In line with a previous study (14), patients were classified into either “BPA responders” or “BPA nonresponders” according to the hemodynamic results at follow-up after their last BPA session. “BPA responders” were defined as mPAP ≤ 30 mmHg and/or a reduction of PVR ≥ 30%. Otherwise, the patients were classified as “BPA nonresponders.”

### Statistical Analysis

Continuous variables are presented as the mean ± standard deviation or median (interquartile range) according to the data distribution. Categorical variables are given as counts (percentages). An independent-sample *t*-test, the Mann–Whitney *U* test, the chi-square test, a paired *t*-test or the Wilcoxon signed rank test was used to compare clinical parameters between groups where appropriate. Two-way analyses of variance were used to compare the DLCO values at the baseline, after the first BPA session and at follow-up with Bonferroni *post hoc* tests for pairwise comparisons. Spearman correlation coefficients were first calculated to identify variables potentially associated with the ΔDLCO. To adjust for potential confounding factors, variables with a Spearman correlation coefficient at *P* < 0.05 and those that were clinically significant were further assessed based on a multivariate linear regression analysis (enter method). A receiver operator characteristic curve analysis was performed to identify the optimal cutoff of the baseline DLCO value and ΔDLCO in predicting unfavorable response to BPA as well as the area under the curve (AUC) of each variable. The DeLong test was used for comparisons of the AUC values. Univariate logistic regression was first performed to identify variables potentially associated with an unfavorable response to BPA. Subsequently, variables with a *P*-value < 0.05 in the univariate models or of clinical significance were included in the multivariate logistic regression model (enter method). A two-sided *P*-value < 0.05 was considered statistically significant. Statistical analyses were performed with SPSS 25.0 (IBM SPSS Corp.; Armonk, NY, USA), Prism GraphPad 8 (GraphPad Software, LaJolla, CA, USA), and MedCalc (MedCalc 19.7.2 version, MedCalc Inc., Mariakerke, Belgium).

## Results

### Baseline Demographics

**Figure 1:**
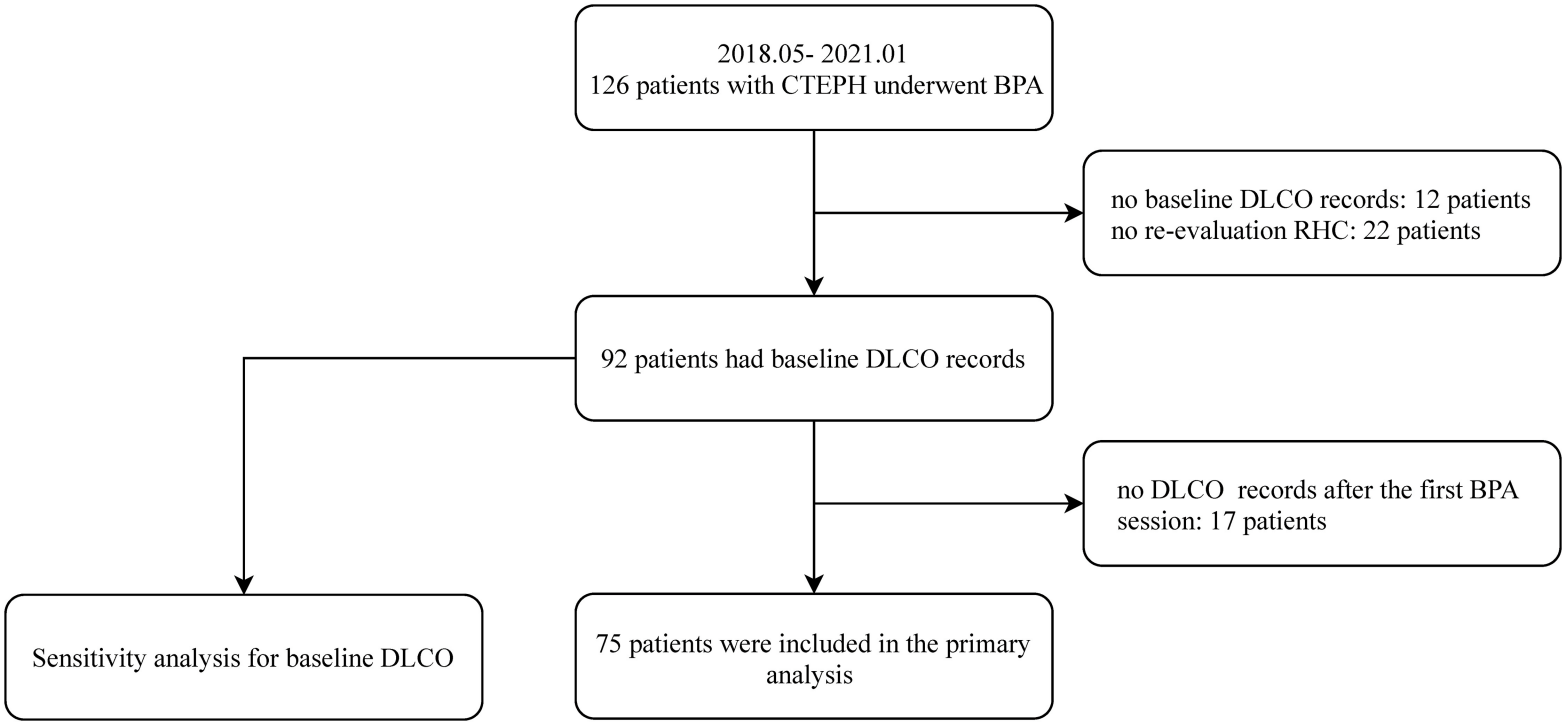
The patient enrollment flow chart. BPA, balloon pulmonary angioplasty; CTEPH, chronic thromboembolic pulmonary hypertension; DLCO, diffusing capacity for carbon monoxide; RHC, right heart catheterization.

As shown in Figure 1, a total of 126 patients with CTEPH underwent BPA between May 2018 and January 2021. Among them, 51 patients were excluded for no baseline DLCO records (*n* = 12), no DLCO records after the first BPA session (*n* = 17) or no reevaluation RHC at follow-up (*n* = 22). Of the 75 included patients, the mean age was 57.5 ± 11.9 years old and 40 (52.6%) were female. On average, the patients underwent 2.2 ± 1.3 BPA sessions with 14.8 ± 9.4 subsegmental pulmonary vessels dilated, and the median time interval from the baseline to reevaluation RHC was 30 weeks (interquartile range, 15–60 weeks). At follow-up, 45 achieved an mPAP ≤ 30 mmHg and/or a reduction of PVR ≥ 30%, whereas 30 patients failed.

### BPA Responders vs. Nonresponders

At the baseline, the BPA responders had a significantly higher DLCO (67 ± 12.8 vs. 59.5 ± 13.5%, *P* = 0.017) than the nonresponders. The rest of the baseline characteristics were comparable between the BPA responders and nonresponders. The details are summarized in Table 1.

**Table 1:** Clinical features of BPA responders and nonresponders at the baseline.

**Variables**	**Responder**	**Non-responder**	***P-*value**
	**(*n* = 45)**	**(*n* = 30)**	
Age, years	57.2 ± 12.2	57.9 ± 11.7	0.810
Female, *n* (%)	24(53.3)	16(53.3)	1.000
BMI, kg/m^2^	24.4 ± 3.3	23.4 ± 3.2	0.190
**WHO FC**			0.296
I or II, *n* (%)	22(48.9)	11(36.7)	
III or IV, *n* (%)	23(51.1)	19(63.3)	
NT-proBNP, ng/L	497.0	906.6	0.086
	(107.2, 1495.5)	(234.1, 2560.5)	
S_a_O_2_, %	91.3 ± 5.7	91.2 ± 3.8	0.284
6MWD, *m*	386.8 ± 96.5	375.3 ± 126.2	0.666
**PH specific drug**			0.182
None	22(48.9)	10(33.3)	
Mono-therapy/ Combination	23(51.1)	20(66.7)	
ERA, *n* (%)	9(20.0)	5(16.7)	0.717
PDE5i, *n* (%)	12(26.7)	12(40.0)	0.225
Riociguat, *n* (%)	8(17.8)	5(16.7)	0.901
Prostacyclins, *n* (%)	0(0)	0(0)	1.000
**Echocardiography**			
LA, mm	33.4 ± 7	35.1 ± 5.7	0.068
LVED, mm	41 ± 5.9	42.1 ± 5.1	0.189
RVED, mm	31.8 ± 5.6	32.8 ± 6.9	0.824
RVED/LVED	0.8 ± 0.2	0.8 ± 0.2	0.534
EF, %	65 ± 6.2	64.9 ± 5.2	0.797
TRV, m/s	4.3 ± 0.7	4.4 ± 0.6	0.650
**Hemodynamics**			
S_v_O_2_, %	69.9 ± 5.3	69.2 ± 5.6	0.619
mRAP, mmHg	8.0 ± 3.2	8.3 ± 3.5	0.756
sPAP, mmHg	93.2 ± 21.5	91.9 ± 18.6	0.790
dPAP, mmHg	30.9 ± 8.9	32.6 ± 8.2	0.402
mPAP, mmHg	51.2 ± 12.2	51.5 ± 11.2	0.957
PAWP, mmHg	9.6 ± 3.1	10.9 ± 3.3	0.116
Cardiac index, L/min.m^2^	3 ± 0.8	2.7 ± 0.8	0.183
PVR, wood units	10.2 ± 4.4	10.0 ± 3.9	0.804
**Cardiopulmonary exercise test**			
VO_2_@Peak, mL/min/kg	12.7 ± 3.5	12.3 ± 2.8	0.615
VO_2_@Peak, % predicted	50.7 ± 14	48.3 ± 13.3	0.455
VE/VCO_2_ slope	49.1 ± 9.8	49 ± 10.1	0.960
**Pulmonary function test**			
FVC, % predicted	91.2 ± 14.2	87.9 ± 22.2	0.138
FEV_1_, % predicted	82.9 ± 15.4	78.6 ± 18.9	0.283
FEV_1_/FVC, %	74.2 ± 6.7	74.1 ± 6.4	0.944
DLCO, % predicted	67.0 ± 12.8	59.5 ± 13.5	**0.017**
DLCO/VA, % predicted	82.8 ± 16.7	76.8 ± 13	0.089

*Data are presented as mean ± standard deviation, median (range) or number (percentage). BPA, balloon pulmonary angioplasty; BMI, body mass index; DLCO, diffusing capacity for carbon monoxide; dPAP, diastolic pulmonary arterial pressure; EF, ejection fraction; ERA, endothelin receptor antagonist; FVC, forced vital capacity; FEV_1_, forced expiratory volume in 1 s; LA, left atrium dimension; LVED, left ventricular end-diastolic diameter; mPAP, mean pulmonary arterial pressure; mRAP, mean right atrial pressure; NT-proBNP, N-terminal pro-brain natriuretic peptide; PAWP, pulmonary arterial wedge pressure; PH, pulmonary hypertension; PDE5i, phosphodiesterase type 5 inhibitors; PVR, pulmonary vascular resistance; RVED, right ventricular end-diastolic diameter; 6 MWD, 6-min walk distance; S_a_O_2_, arterial oxygen saturation; sPAP, systolic pulmonary arterial pressure; S_V_O_2_, mixed venous oxygen saturation; TRV, tricuspid regurgitation velocity; VA, alveolar ventilation; VE/VCO_2_ slope, minute ventilation/carbon dioxide output slope; VO_2_@Peak, peak oxygen consumption; WHO FC, World Health Organization functional class. Bold values mean their P value < 0.050*.

During the first BPA session, we found that the number of dilated subsegmental pulmonary vessels was comparable between the BPA responders and nonresponders (5.9 ± 1.7 vs. 5.9 ± 2.2, *P* = 0.880). We also calculated the proportion of lower lobe vessels (A6–A10) (7) in the dilated subsegmental pulmonary vessels during the first BPA session for each patient. The results showed that the operators mainly dilated lower lobe vessels in both the BPA responder (64.1 ± 20.% of the dilated subsegmental pulmonary vessels) and nonresponder (55.9 ± 25.1% of the dilated subsegmental pulmonary vessels) groups during the first BPA session; moreover, the proportion of lower lobe vessels was comparable between the two groups (*P* = 0.114). During the first BPA session, BPA nonresponders had a higher incidence of hemoptysis (6.7 vs. 2.2%, *P* = 0.718) than did responders, although the difference did not reach statistical significance (shown in Table 2).

**Table 2:** Clinical features of BPA responders and nonresponders at follow-up.

**Variables**	**Responder**	**Non-responder**	***P-*value**
	**(*n* = 45)**	**(*n* = 30)**	
Number of BPA sessions	2.5 ± 1.4	1.8 ± 1.0	**0.027**
Number of dilated subsegmental pulmonary vessels	16.7 ± 10.4	11.8 ± 6.9	**0.046**
Time interval, weeks	42.5 ± 27.9	31.4 ± 24.3	0.124
**WHO FC**			0.470
I or II, *n* (%)	41(91.1)	25(83.3)	
III or IV, *n* (%)	4(8.9)	5(16.7)	
NT-proBNP, ng/L	103.0	339.6	**0.018**
	(49.5, 246.5)	(77.1, 816.1)	
S_a_O_2_, %	93.8 ± 2.4	91.8 ± 6.0	**0.021**
6MWD, *m*	440.0 ± 84.7	431.9 ± 95.5	0.745
**Echocardiography**			
LA, mm	34.2 ± 6.1	35.5 ± 8.5	0.454
LVED, mm	44.4 ± 4.7	43.2 ± 4.5	0.313
RVED, mm	28.0 ± 4.8	30.3 ± 7	0.162
RVED/LVED	0.6 ± 0.1	0.7 ± 0.2	0.092
EF, %	66.3 ± 5	62.7 ± 6.1	**0.026**
TRV, m/s	3.7 ± 0.7	4.1 ± 0.7	0.492
**Hemodynamics**			
S_v_O_2_, %	72.6 ± 4.8	70.8 ± 5.3	0.141
mRAP, mmHg	6.7 ± 3.1	7.4 ± 3.2	0.345
sPAP, mmHg	62.9 ± 21.6	81.1 ± 21.3	**<0.001**
dPAP, mmHg	21.8 ± 5.4	28.4 ± 7.4	**<0.001**
mPAP, mmHg	35.3 ± 9.4	45.5 ± 10.8	**<0.001**
PAWP, mmHg	10.5 ± 3.6	10.1 ± 3.7	0.632
Cardiac index, L/min.m^2^	3.3 ± 0.6	3.3 ± 1.2	0.234
PVR, wood units	5.1 ± 2.3	8.3 ± 2.9	**<0.001**
**Cardiopulmonary exercise test**			
VO_2_@Peak, ml/min/kg	14.8 ± 3.6	13.8 ± 3.5	0.282
VO_2_@Peak, % predicted	60.5 ± 15.5	54.3 ± 15.9	0.104
VE/VCO_2_ slope	41.4 ± 8.3	44.6 ± 7.8	0.104
**Pulmonary function test**			
FVC, % predicted	92.5 ± 14.3	88.1 ± 20.6	0.225
FEV_1_, % predicted	84.7 ± 15.7	77.6 ± 19.5	0.088
FEV_1_/FVC, %	74.9 ± 5.6	72.3 ± 7.2	0.091
DLCO, % predicted	67.3 ± 13.9	63.1 ± 13.2	0.205
DLCO/VA, % predicted	80.2 ± 16	78.2 ± 12.3	0.859
Complications^*^			
Pulmonary vessel extravasation, *n* (%)	0.0(0.0)	0.0(0.0)	1.000
Hemoptysis, *n* (%)	1(2.2)	2(6.7)	0.718
Reperfusion pulmonary edema, *n* (%)	0.0(0.0)	0.0(0.0)	1.000

*Data are presented as mean ± standard deviation, median (range) or number (percentage). BPA, balloon pulmonary angioplasty; DLCO, diffusing capacity for carbon monoxide; dPAP, diastolic pulmonary arterial pressure; EF, ejection fraction; FVC, forced vital capacity; FEV_1_, forced expiratory volume in 1 s; LA, left atrium dimension; LVED, left ventricular end-diastolic diameter; mPAP, mean pulmonary arterial pressure; mRAP, mean right atrial pressure; NT-proBNP, N-terminal pro-brain natriuretic peptide; PAWP, pulmonary arterial wedge pressure; PVR, pulmonary vascular resistance; RVED, right ventricular end-diastolic diameter; 6 MWD, 6-min walk distance; S_a_O_2_, arterial oxygen saturation; sPAP, systolic pulmonary arterial pressure; S_V_O_2_, mixed venous oxygen saturation; TRV, tricuspid regurgitation velocity; VA, alveolar ventilation; VE/VCO_2_ slope, minute ventilation/carbon dioxide output slope; VO_2_@Peak, peak oxygen consumption; WHO FC, World Health Organization functional class*.

* *Complications occurred during the first BPA session. Time interval is the interval between baseline and follow-up. Bold values mean their P value < 0.050*.

At follow-up, responders underwent more BPA sessions (2.5 ± 1.4 vs. 1.8 ± 1, *P* = 0.027) and had more dilated subsegmental pulmonary vessels (16.7 ± 10.4 vs. 11.8 ± 6.9, *P* = 0.046) than BPA nonresponders. The time interval from the baseline to follow-up tended to be longer in BPA responders (42.5 ± 27.9 vs. 31.4 ± 24.3 weeks, *P* = 0.124). Reasonably, BPA responders had lower NT-proBNP levels, higher arterial oxygen saturation, and more favorable parameters derived from echocardiography and RHC than nonresponders, although these parameters were comparable at the baseline between the BPA responders and nonresponders. The details are summarized in Table 2.

### Effect of BPA on the DLCO

**Figure 2:**
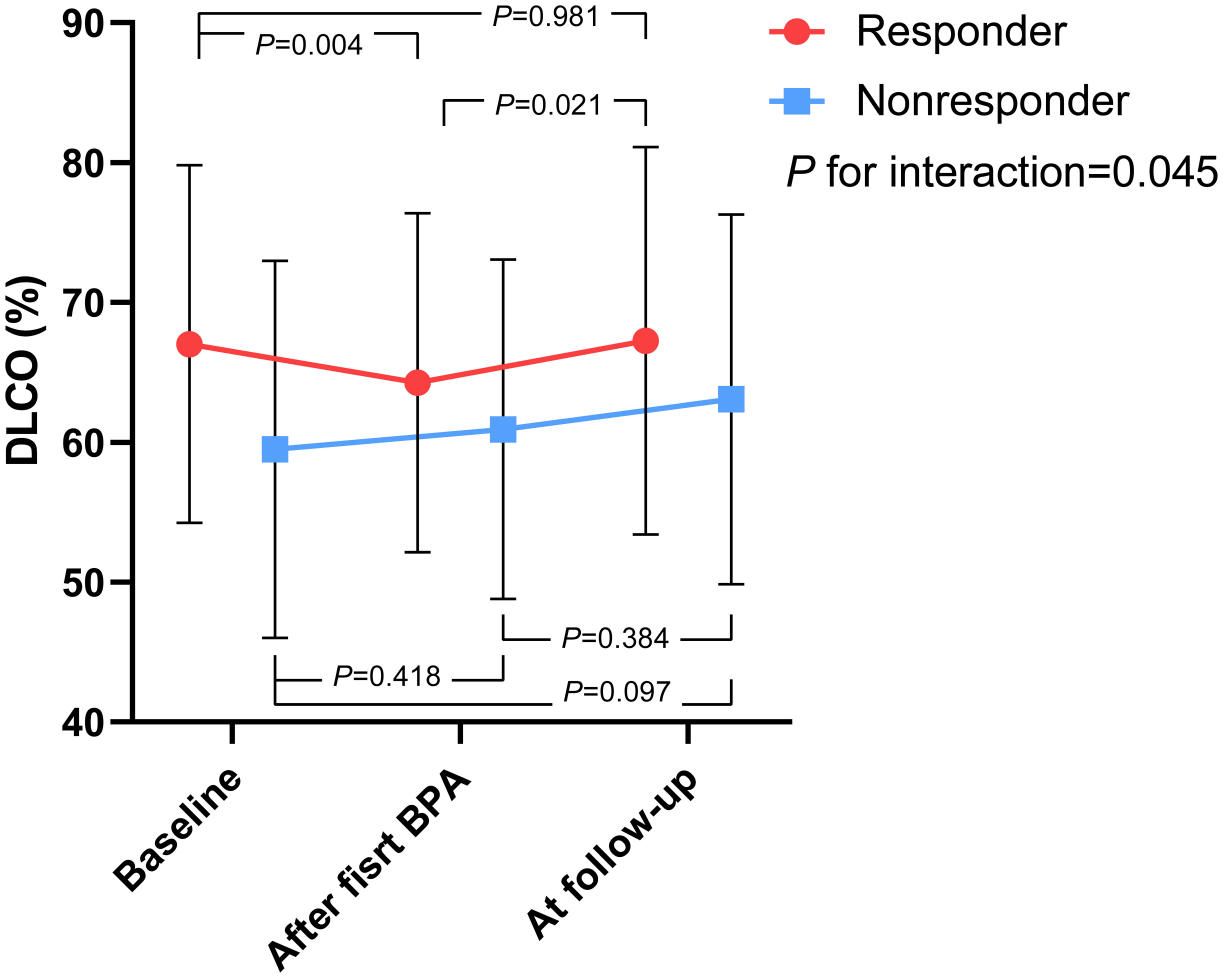
The time course of DLCO values during the BPA procedure, stratified by BPA responders and nonresponders. *P* for interaction represents the interaction between the outcome of BPA and time course as within-subjects effects. BPA, balloon pulmonary angioplasty; DLCO, diffusing capacity for carbon monoxide.

As shown in Figure 2, the time course of DLCO values during the BPA procedure differed between the BPA responders and nonresponders. For the BPA responders, the DLCO decreased within 7 days after the first BPA session (after the first BPA vs. the baseline: 64.3 ± 12.1 vs. 67 ± 12.8%, *P* = 0.004) and then returned to a level similar to the baseline at follow-up (follow-up vs. baseline: 67.3 ± 13.9 vs. 67 ± 12.8%, *P* = 0.981). For the BPA nonresponders, the DLCO tended to increase within 7 days after the first BPA session (after first BPA vs. baseline: 60.9 ± 12.1 vs. 59.5 ± 13.5%, *P* = 0.418), and this trend persisted at follow-up (follow-up vs. baseline: 63.1 ± 13.2 vs. 59.5 ± 13.5%, *P* = 0.097).

### Parameters Associated With ΔDLCO

The Spearman correlation coefficient showed that the baseline DLCO value (*r* = −0.340, *P* = 0.003) was the only variable associated with ΔDLCO among the various demographic, echocardiographic, and hemodynamic parameters. After adjusting for age, sex, body mass index, mPAP, and forced vital capacity percentage change between the baseline and after the first BPA session, the baseline DLCO values were still associated with the ΔDLCO (standardized β coefficient = −0.535, *P* < 0.001). The details are summarized in Table 3.

**Table 3:** Correlation between ΔDLCO and various variables at the baseline or the change of these variables after the first BPA session.

**Variables**	**Coefficient**	***P* value**	**Adjusted**	***P* value**
			**coefficient**	
Age	0.096	0.418	0.094	0.385
Female	0.051	0.665	−0.078	0.492
BMI	0.133	0.257	0.221	0.065
WHO FC	0.073	0.531		
NT-proBNP	0.072	0.539		
S_a_O_2_	0.012	0.918		
6MWD	0.043	0.720		
Targeted therapy at baseline	0.127	0.279		
LA	0.196	0.092		
LVED	0.121	0.302		
RVED	−0.129	0.271		
RVED/LVED	−0.171	0.143		
EF	−0.048	0.684		
TRV	0.078	0.508		
S_v_O_2_	0.004	0.972		
mRAP	0.087	0.460		
mPAP	0.024	0.839		
PAWP	0.018	0.883		
Cardiac index	0.041	0.729		
PVR	−0.060	0.611		
VO_2_@Peak	−0.213	0.069		
VE/VCO_2_ slope	−0.010	0.932		
FVC	−0.046	0.692		
FEV_1_	−0.060	0.609		
FEV_1_/FVC	−0.022	0.850		
DLCO	−0.340	**0.003**	−0.535	**<0.001**
DLCO/VA	−0.191	0.101		
Number of dilated subsegmental pulmonary vessels during the first BPA session	<0.001	0.998		
ΔNT-proBNP	−0.181	0.119		
Δ6MWD	−0.185	0.164		
ΔLA	−0.101	0.390		
ΔLVED	0.054	0.648		
ΔRVED	0.048	0.682		
ΔEF	0.169	0.147		
ΔTRV	0.004	0.971		
ΔmPAP	0.095	0.415	0.129	0.226
ΔVO_2_@Peak	0.093	0.432		
ΔVE/VCO_2_ slope	−0.013	0.917		
ΔFVC	0.151	0.197	0.077	0.473
ΔFEV_1_	0.170	0.145		
ΔFEV_1_/FVC	0.091	0.436		

*Adjusted coefficient used multivariate linear regression to adjust for age, sex, BMI, ΔFVC, DLCO at the baseline, LA, and ΔmPAP; ΔDLCO, (DLCO after the first BPA session—DLCO before the first BPA session)/DLCO before the first BPA session; BMI, body mass index; BPA, balloon pulmonary angioplasty; DLCO, diffusing capacity for carbon monoxide; dPAP, diastolic pulmonary arterial pressure; EF, ejection fraction; FVC, forced vital capacity; FEV_1_, forced expiratory volume in 1 s; LA, left atrium dimension; LVED, left ventricular end-diastolic diameter; mPAP, mean pulmonary arterial pressure; mRAP, mean right atrial pressure; NT-proBNP, N-terminal pro-brain natriuretic peptide; PAWP, pulmonary arterial wedge pressure; PVR, pulmonary vascular resistance; RVED, right ventricular end-diastolic diameter; 6 MWD, 6-min walk distance; S_a_O_2_, arterial oxygen saturation; sPAP, systolic pulmonary arterial pressure; S_V_O_2_, mixed venous oxygen saturation; TRV, tricuspid regurgitation velocity; VA, alveolar ventilation; VE/VCO_2_ slope, minute ventilation/carbon dioxide output slope; VO_2_@Peak, peak oxygen consumption; WHO FC, World Health Organization functional class. Bold values mean their P value < 0.050*.

### Cutoff Value for DLCO and ΔDLCO in Predicting Unfavorable BPA Response

**Figure 3:**
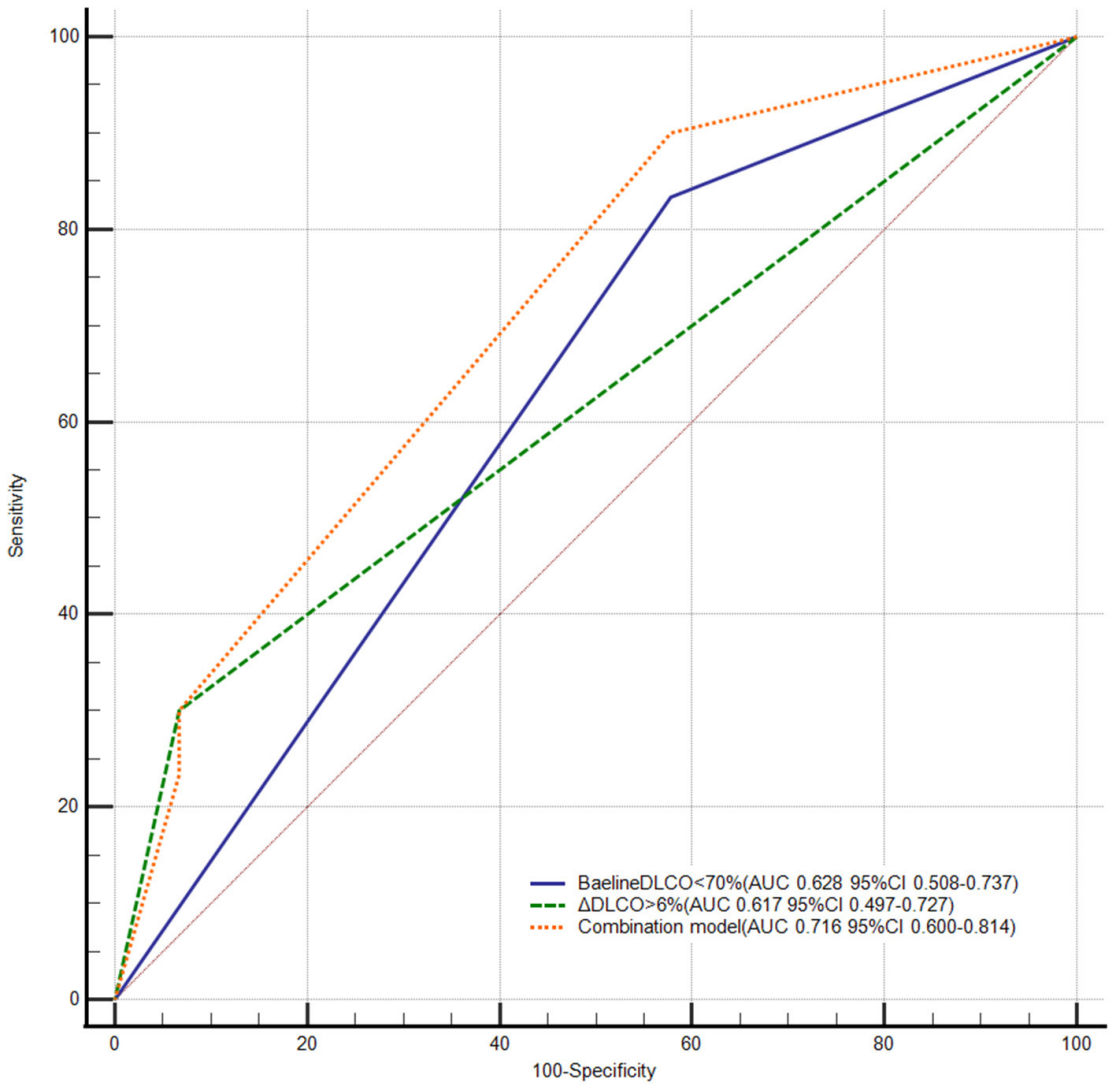
Receiver operator characteristic curve of DLCO in predicting unfavorable response to BPA. BPA, balloon pulmonary angioplasty; DLCO, diffusing capacity for carbon monoxide; ΔDLCO, the percent change of DLCO between the baseline and within 7 days after the first BPA session. DeLong test pairwise comparison: Baseline < DLCO < 70% vs. the combination model *P* = 0.041; ΔDLCO > 6% vs. the combination model *P* = 0.010; ΔDLCO > 6% vs. baseline DLCO < 70% *P* = 0.871.

The receiver operator characteristic curve showed that the best cutoff value in predicting unfavorable BPA response was 70% for DLCO values at the baseline (AUC: 0.628, 95% CI: 0.508–0.737) and 2.4% for ΔDLCO (AUC: 0.683, 95% CI: 0.555–0.812). Previous studies showed that the mean percentage difference between two repeat DLCO measurements ranged from 2.5 to 4.4% in patients without ventilatory dysfunction (15, 16). Meanwhile, our data showed that the mean percentage difference between two repeat measurements of DLCO was 3.5% in patients with pulmonary hypertension [*n* = 32, the time interval between two repeat measurements should be <7 days, and treatment should not be changed between two repeated measurements (e.g., did not change targeted drug or did not undergo BPA)]. To maximize the generalizability of our conclusion, we reset the cutoff value of ΔDLCO to 6% (AUC: 0.617, 95% CI: 0.497–0.727) (Supplementary Table 1). Accordingly, 51 and 24 patients were classified as DLCO < 70% and DLCO ≥ 70%, respectively; 64 and 11 patients were classified as ΔDLCO ≤ 6% and ΔDLCO > 6%, respectively; and nine patients had DLCO < 70% and ΔDLCO > 6%. Although the AUCs were comparable between the baseline DLCO values and ΔDLCO (*P* = 0.871), we found that the combination of these two variables (AUC: 0.716, 95% CI: 0.600–0.814) demonstrated a superior AUC than either of the two variables alone (*P* = 0.041, compared to the baseline DLCO values; *P* = 0.010, compared to the ΔDLCO) (Figure 3).

**Figure 4:**
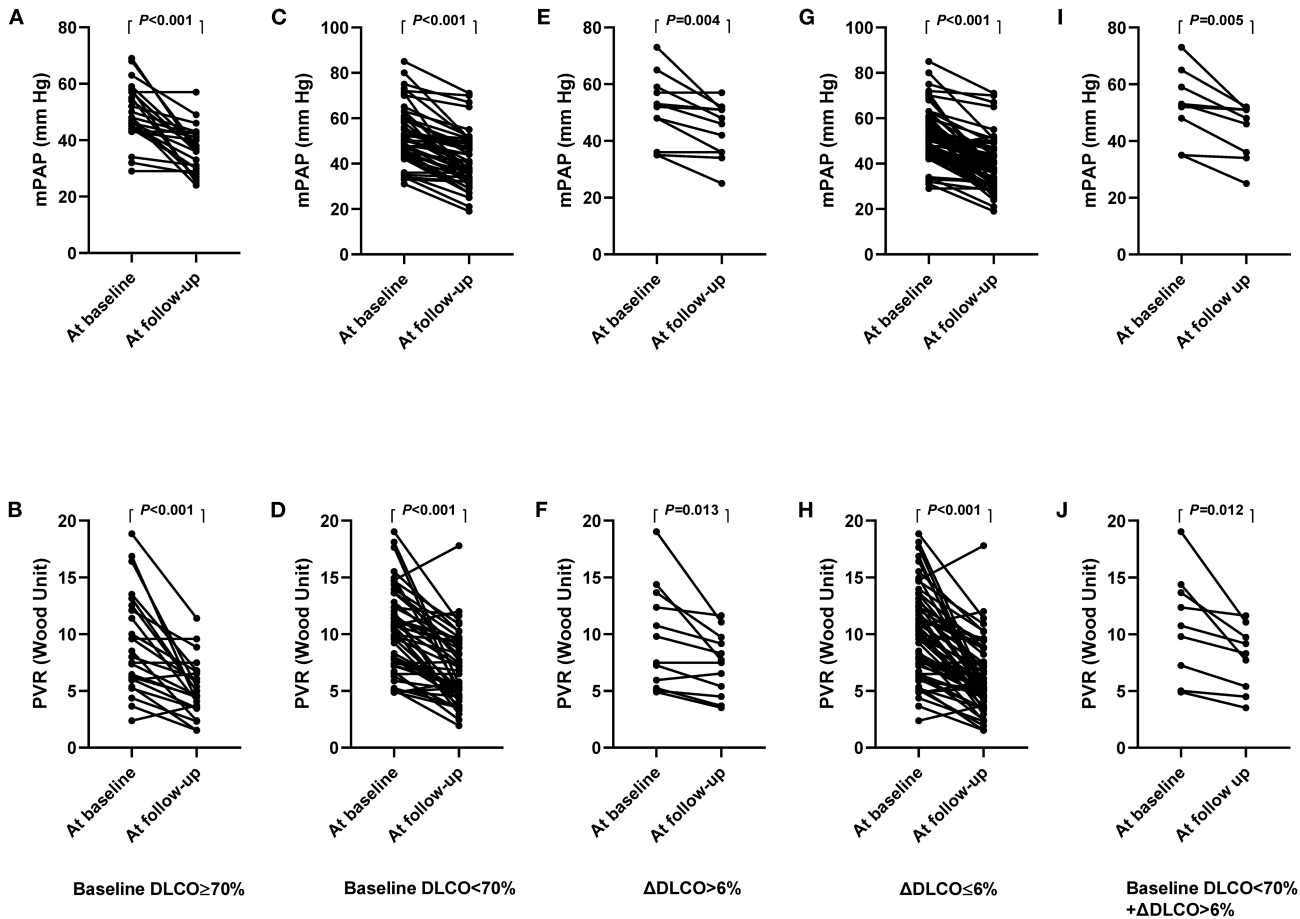
**(A–J)**, hemodynamics at the baseline and follow-up, stratified by baseline DLCO and ΔDLCO. mPAP **(A)** and PVR **(B)** in patients with baseline DLCO ≥ 70%. mPAP **(C)** and PVR **(D)** in patients with baseline DLCO < 70%. mPAP **(E)** and PVR **(F)** in patients with ΔDLCO > 6%. mPAP **(G)** and PVR **(H)** in patients with ΔDLCO ≤ 6%. mPAP **(I)** and PVR **(J)** in patients with baseline DLCO < 70% and ΔDLCO > 6%. DLCO, diffusing capacity for carbon monoxide; ΔDLCO, the percent change of DLCO between the baseline and within 7 days after the first BPA session. mPAP, mean pulmonary arterial pressure; PVR, pulmonary vascular resistance.

The clinical characteristics of patients with a baseline DLCO < 70% and DLCO ≥ 70% at the baseline and at follow-up are summarized in Supplementary Tables 2, 3. Compared with the baseline, mPAP and PVR decreased at follow-up in both the baseline DLCO < 70% and DLCO ≥ 70% groups (Figure 4). However, patients with a baseline DLCO ≥ 70% had a significantly higher proportion of BPA responders than those with a DLCO < 70%, although BPA sessions, dilated vessels, and the time interval between the baseline and follow-up were comparable between the two groups (Supplementary Table 3). Similar results were also observed in patients with ΔDLCO ≤ 6% and ΔDLCO > 6% (Figure 4, Supplementary Tables 4, 5).

### Predictors of Unfavorable Response to BPA

Univariate logistic analysis showed that a baseline DLCO < 70%, ΔDLCO > 6%, BPA session number and dilated pulmonary subsegment number were associated with the BPA response (shown in Table 4). Given their high collinearity with the number of BPA sessions, the number of dilated pulmonary subsegments (*r* = 0.911, *P* < 0.001) and the time interval between the baseline and follow-up (*r* = 0.730, *P* < 0.001) were excluded from the multivariate logistic regression. After adjusting for the number of BPA sessions, the baseline DLCO < 70% (OR: 4.585, 95% CI: 1.021–20.596, *P* = 0.047) and ΔDLCO > 6% (OR: 3.666, 95% CI, 1.094–12.290, *P* = 0.035) were still associated with unfavorable responses to BPA. Further adjustment for the WHO FC, NT-proBNP, and hemodynamic parameters did not attenuate the statistical significance of a baseline DLCO < 70% and ΔDLCO > 6% (shown in Table 5).

**Table 4:** Univariate logistic regression analyses for poor hemodynamic response to BPA.

**Variable**	**OR**	**95%CI**	***P*-value**
Age	1.005	0.966–1.045	0.807
Female	1.000	0.396–2.524	1.000
WHO FC	1.652	0.642–4.251	0.298
Ln(NT-proBNP)	1.321	0.952–1.833	0.096
6MWD	0.999	0.995–1.003	0.661
None/ Medicine treatment	1.913	0.734–4.987	0.184
LVED	1.037	0.954–1.128	0.394
RVED/LVED	1.187	0.125–11.241	0.881
EF	0.998	0.921–1.081	0.954
TRV	1.185	0.576–2.435	0.645
S_v_O_2_	0.977	0.896–1.064	0.589
mRAP	1.023	0.889–1.177	0.752
dPAP	1.024	0.970–1.081	0.398
mPAP	1.002	0.963–1.042	0.916
PAWP	1.146	0.981–1.340	0.086
Cardiac index	0.667	0.368–1.209	0.182
PVR	0.984	0.880–1.101	0.785
FEV_1_/FVC	0.993	0.942–1.045	0.776
Baseline DLCO < 70%	3.654	1.183–11.286	**0.024**
ΔDLCO>6%	5.091	1.226–21.138	**0.025**
DLCO/VA %predicted	0.974	0.944–1.006	0.107
VO_2_@Peak	0.963	0.833–1.113	0.610
VE/VCO_2_ slope	0.999	0.952–1.047	0.960
Number of BPA sessions	0.607	0.397–0.927	**0.021**
Number of dilated subsegmental pulmonary vessels	0.937	0.883–0.994	**0.032**
Time interval	0.984	0.966–1.002	0.083

*Baseline variables were included in analysis. BPA, balloon pulmonary angioplasty; dPAP, diastolic pulmonary arterial pressure; DLCO, diffusing capacity for carbon monoxide; ΔDLCO, (DLCO after the first BPA session—DLCO before the first BPA session)/DLCO before the first BPA session; EF, ejection fraction; FVC, forced vital capacity; FEV_1_, forced expiratory volume in 1 s; LVED, left ventricular end-diastolic diameter; mPAP, mean pulmonary arterial pressure; mRAP, mean right atrial pressure; NT-proBNP, N-terminal pro-brain natriuretic peptide; PAWP, pulmonary arterial wedge pressure; PVR, pulmonary vascular resistance; RVED, right ventricular end-diastolic diameter; 6 MWD, 6-min walk distance; S_v_O_2_, mixed venous oxygen saturation; TRV, tricuspid regurgitation velocity; VA, alveolar ventilation; VE/VCO_2_ slope, minute ventilation/carbon dioxide output slope; VO_2_@Peak, peak oxygen consumption; WHO FC, World Health Organization functional class. Time interval is the interval between baseline and follow-up. Bold values mean their P value < 0.050*.

**Table 5:** Multivariate logistic regression analyses for poor hemodynamic response to BPA.

**Model**	**Variable**	**OR**	**95%CI**	***P*-value**
1	ΔDLCO>6%	4.585	1.021–20.596	**0.047**
	DLCO < 70%	3.666	1.094–12.290	**0.035**
	Number of BPA sessions	0.589	0.372–0.932	**0.024**
2	ΔDLCO>6%	4.619	1.011–21.113	**0.048**
	DLCO < 70%	3.496	1.038–11.782	**0.043**
	Number of BPA sessions	0.591	0.374–0.935	**0.025**
	WHO FC	1.391	0.486–3.985	0.539
3	ΔDLCO>6%	4.522	0.972–21.039	**0.049**
	DLCO < 70%	3.749	1.092–12.871	**0.036**
	Number of BPA sessions	0.550	0.339–0.894	**0.016**
	Ln(NT-proBNP)	1.428	0.974–2.091	0.068
4	ΔDLCO>6%	4.612	1.021–20.834	**0.047**
	DLCO < 70%	3.620	1.062–12.334	**0.040**
	Number of BPA sessions	0.590	0.373–0.935	**0.025**
	SvO_2_	0.994	0.900–1.097	0.905
5	ΔDLCO>6%	4.572	1.017–20.561	**0.048**
	DLCO < 70%	3.625	1.071–12.275	**0.038**
	Number of BPA sessions	0.587	0.370–0.931	**0.024**
	mPAP	1.003	0.960–1.048	0.889
6	ΔDLCO>6%	4.545	1.012–20.416	**0.048**
	DLCO < 70%	3.914	1.122–13.650	**0.032**
	Number of BPA sessions	0.590	0.370–0.939	**0.026**
	PVR	0.969	0.851–1.104	0.638
7	ΔDLCO>6%	4.763	1.024–22.160	**0.047**
	DLCO < 70%	4.292	1.181–15.607	**0.027**
	Number of BPA sessions	0.543	0.328–0.899	**0.018**
	PAWP	1.170	0.969–1.414	0.102
8	ΔDLCO>6%	5.710	1.191–27.364	**0.029**
	DLCO < 70%	3.557	1.060–11.939	**0.040**
	Number of BPA sessions	0.605	0.384–0.952	**0.030**
	Cardiac index	0.574	0.288–1.143	0.114

*BPA, balloon pulmonary angioplasty; DLCO, diffusing capacity for carbon monoxide; ΔDLCO, (DLCO after the first BPA session—DLCO before the first BPA session)/DLCO before the first BPA session; mPAP, mean pulmonary arterial pressure; NT-proBNP, N-terminal pro-brain natriuretic peptide; PAWP, pulmonary arterial wedge pressure; PVR, pulmonary vascular resistance; S_v_O_2_, mixed venous oxygen saturation; WHO FC, World Health Organization functional class. Bold values mean their P value < 0.050*.

### Sensitivity Analysis

As shown in Figure 1, 92 patients had baseline DLCO records. We reassessed the predictive value of the baseline DLCO < 70% in these patients. The results showed that the baseline DLCO < 70% was still an independent predictor of unfavorable response to BPA (Supplementary Tables 6–11).

We also compared the baseline characteristics of the included and excluded patients. Except for younger age and better WHO FC in the included patients, exercise tolerance and echocardiographic and hemodynamic parameters were comparable between the included and excluded patients (Supplementary Table 12).

## Discussion

In the current study, we demonstrated, for the first time, that the baseline DLCO < 70% and ΔDLCO > 6% were independent predictors of an unfavorable response to BPA. More importantly, the combination of the baseline DLCO < 70% and ΔDLCO > 6% demonstrated a superior predictive ability than either of these two variables alone.

### Effect of BPA on the DLCO

We observed the opposite trend in the changes in the DLCO after the first BPA session between BPA responders and nonresponders, although the two groups had similar numbers of dilated subsegmental pulmonary vessels, and both mainly had dilated lower lobe vessels. For BPA responders, the DLCO values decreased after the first BPA session, which was consistent with the work of Akizuki et al. (7), who suggested that, when dilating lower lobe vessels, the elevation of ventilation is disproportional to hemodynamic improvement, which exacerbates the ventilation/perfusion mismatch and results in decreased DLCO (7). In contrast, DLCO values in nonresponders remained unchanged and even tended to increase after the first BPA session (60.9 ± 12.1 vs. 59.5 ± 13.5%, *P* = 0.418). We provide our own hypothesis for this unique phenomenon. BPA nonresponders had lower baseline DLCO values than responders (Table 1), which indicated that their microvasculopathy was also more advanced. Therefore, nonresponders might have a more dramatic redistribution of pulmonary blood flow from nonoccluded to newly dilated areas after BPA (vascular steal phenomenon) than responders, which has been observed in patients after undergoing PEA and is thought to be related to microvasculopathy (17, 18). Consequently, newly dilated vessels in nonresponders suffered from significantly higher shear stress and wall tension than did responders. Coupled with potential vascular wall damage caused by BPA, red blood cells might leak into alveolar airspaces, which could be overt (hemoptysis) or subclinical. Our hypothesis was further supported by the observation that nonresponders tended to have a higher incidence of hemoptysis than did responders during the first BPA session (6.7 vs. 2.2%, *P* = 0.718). Previous studies have revealed that alveolar hemorrhage could lead to elevation of DLCO values (19, 20). Taken together, we suggest that the effect of alveolar hemorrhage on DLCO outweighed that of ventilation/perfusion mismatch in BPA nonresponders, which led to the elevation of DLCO values within 7 days after the first BPA session.

We also performed a comprehensive investigation to screen variables potentially associated with the ΔDLCO and spearman coefficient showed that the baseline DLCO value was the only variable associated with the ΔDLCO among the various demographic, echocardiographic, and hemodynamic parameters (Table 3). This association persisted even after adjusting for age, sex, body mass index, and mPAP and forced vital capacity percent change between the baseline and after the first BPA procedure. We thought that the relationship between the baseline DLCO and ΔDLCO might be explained by the fact that they were both external manifestations of microvasculopathy, which further supported our aforementioned hypothesis.

Regarding the long-term effect of BPA on the DLCO (i.e., from “within 7 days after the first BPA session” to “at follow-up),” we found an increasing DLCO trend in both BPA responders and nonresponders. Akizuki et al. (7) also found that the DLCO would decrease in early BPA sessions, which mainly dilated lower lobe vessels and would recover in later BPA sessions, which mainly dilated upper-middle lobe vessels. They suggested that this may be attributed to intervention in the upper-middle lung field, which increases blood flow in these areas and subsequently attenuates ventilation/perfusion mismatch. Similarly, the proportion of upper-middle lobes in subsequent BPA sessions gradually increased in our center (Supplementary Figure S1).

### Predictive Value of the Baseline DLCO and ΔDLCO

We found that both the baseline DLCO < 70% and ΔDLCO > 6% could independently predict unfavorable responses to BPA. More importantly, the combination of the baseline DLCO < 70% and ΔDLCO > 6% demonstrated a superior predictive ability than either of these two variables alone, which might also be explained by their associations with microvasculopathy. Previous studies showed that concomitant microvasculopathy had a strong influence on the outcome of PEA (2, 21). Moreover, microvasculopathy is irreversible, and patients with advanced microvascular impairment can still suffer from exercise intolerance and hypoxia even after normalization of pulmonary hemodynamics by PEA or BPA (22, 23). In the present study, the DLCO was comparable between the baseline and follow-up, which was consistent with previous studies (24, 25) and suggested a limited effect of BPA on microvasculopathy.

In addition to the DLCO found in the present study, Tsuji et al. reported that diastolic PAP could also predict residual PH after BPA (6). These authors also thought that the underlying mechanism was attributable to the development of pulmonary microvasculopathy secondary to long-standing pulmonary hypertension. Additionally, Taniguchi et al. reported that poor subpleural perfusion, which also reflects microvasculopathy in patients with inoperable CTEPH, could also predict failure of BPA (14). Compared with diastolic PAP and subpleural perfusion, the DLCO might be of more clinical significance due to its non-invasiveness and wide availability.

## Limitation

The current study had several limitations. First, 51 patients were excluded from the primary analysis due to the lack of baseline DLCO records, lack of RHC reevaluation at follow-up, or lack of DLCO records after the first BPA session. However, we performed a sensitivity analysis for the baseline DLCO < 70% in 92 patients, and the results remained unchanged. We also found that the baseline characteristics of the included and excluded patients were comparable. Therefore, the exclusion of these patients should not undermine the conclusions of the current study. Second, the association between the ΔDLCO and microvasculopathy was not pathologically confirmed; thus, further animal or clinical studies are needed. Third, direct evidence for alveolar hemorrhage was not available due to the retrospective nature of the present study. Moreover, potentially subclinical alveolar hemorrhage increases the difficulty of quantifying its impact on the DLCO. Fourth, the number of BPA sessions and dilated vessels were both relatively small for the BPA responders and nonresponders, which means that the nonresponders in our study may convert into responders in future BPA sessions. Furthermore, mPAP and PVR were also decreased significantly in patients with the baseline DLCO < 70% and/or ΔDLCO > 6% after multiple BPA sessions. Thus, the baseline DLCO < 70% and/or ΔDLCO > 6% may not represent a contraindication for BPA. Clinicians could anticipate that patients with the baseline DLCO < 70% and/or ΔDLCO > 6% need more BPA sessions to achieve satisfactory results.

## Conclusion

The baseline DLCO < 70% and ΔDLCO > 6% were both independent predictors of unfavorable response to BPA. Measuring the DLCO dynamically could facilitate the identification of patients who might have an unsatisfactory hemodynamic response to BPA.

## Data Availability Statement

The original contributions presented in the study are included in the article/Supplementary Material, further inquiries can be directed to the corresponding authors.

## Ethics Statement

The studies involving human participants were reviewed and approved by the Ethics Committee of Fuwai Hospital. The patients/participants provided their written informed consent to participate in this study.

## Author Contributions

ZL and ZZ contributed to the conception of the study and guarantors of the paper, taking responsibility for the integrity of the work as a whole, from inception to a published article. YZ and XL wrote the manuscript. QZh, QZe, TY, QJ, LY, AD, XM, JL, and CA contributed to data collection. ZL, CX, and QL contributed to the acquisition of funding. All authors contributed to data analysis and interpretation, critically reviewed the manuscript for intellectual content, and had final responsibility for the decision to submit for publication.

## Funding

This research article was supported by Beijing Municipal Science and Technology Project [Z181100001718200]; Beijing Municipal Natural Science Foundation [7202168]; CAMS Innovation Fund for Medical Sciences (CIFMS) [2020-I2M-C&T-B-055, 2021-I2M-C&T-B-032]; Double First-Class Discipline Construction Fund of Peking Union Medical College and Chinese Academy of Medical Sciences [2019E-XK04-02]; the Capital's Funds for Health Improvement and Research (CFH) [2020-2-4033, 2020-4-4035]; the Youth Fund of Zhongshan Hospital, Fudan University [Grant No. 2021-016].

## Conflict of Interest

The authors declare that the research was conducted in the absence of any commercial or financial relationships that could be construed as a potential conflict of interest.

## Publisher's Note

All claims expressed in this article are solely those of the authors and do not necessarily represent those of their affiliated organizations, or those of the publisher, the editors and the reviewers. Any product that may be evaluated in this article, or claim that may be made by its manufacturer, is not guaranteed or endorsed by the publisher.

## Abbreviations

BPA: Balloon pulmonary angioplasty
CTEPH: chronic thromboembolic pulmonary hypertension
DLCO: diffusing capacity for carbon monoxide
ΔDLCO: Percent change of DLCO between the baseline and within 7 days after the first BPA session
EF: ejection fraction
mPAP: mean pulmonary arterial pressure
NT-proBNP: N-terminal pro-brain natriuretic peptide
PEA: pulmonary endarterectomy
PVR: pulmonary vascular resistance
RHC: right heart catheterization
6MWD: 6-min walk distance
S_V_O_2_: mixed venous oxygen saturation
VE/VCO_2_ slope: minute ventilation/ carbon dioxide output slope
VO_2_@Peak: peak oxygen consumption
WHO FC: World Health Organization functional class.
