# Supplementary material for: Diffusing Capacity for Carbon Monoxide Predicts Response to Balloon Pulmonary Angioplasty in Patients With Inoperable Chronic Thromboembolic Pulmonary Hypertension
Source: Front Cardiovasc Med. 2021 Dec 2;8:762267. doi: 10.3389/fcvm.2021.762267 (PMC8674470; doi:10.3389/fcvm.2021.762267)
Supplement: Supplementary file 1 [file Table_1.docx]

Table S1 Accuracy of DLCO<70%, ΔDLCO>6% and combination of both

| Variable | AUC | 95% CI | Sensitivity | Specificity | Youden index | *P* value |
| --- | --- | --- | --- | --- | --- | --- |
| DLCO<70% | 0.628 | 0.508-0.737 | 83.33 | 42.22 | 0.256 | **0.012** |
| ΔDLCO>6% | 0.617 | 0.497-0.727 | 30 | 93.33 | 0.233 | **0.012** |
| Combination | 0.716 | 0.600-0.814 | 90 | 42.22 | 0.322 | **<0.0001** |

AUC, area under curve, DLCO, Diffusing capacity for carbon monoxide; ΔDLCO, (DLCO after first BPA session- DLCO before first BPA session)/ DLCO before first BPA session. Bold values mean their *P* value < 0.050.

Table S2 Clinical Features of Patients with DLCO<70% and DLCO≥ 70% at Baseline.

| Variables | DLCO≥ 70% (n=24) | DLCO<70% (n=51) | *P* value |
| --- | --- | --- | --- |
| Age, years | 54.7±13.8 | 58.8±10.8 | 0.171 |
| Female, n (%) | 8(33.3) | 32(62.7) | **0.017** |
| BMI, kg/m^2^ | 25.7±3.2 | 23.2±3.0 | **0.001** |
| WHO FC |  |  | 0.224 |
| I or II, n (%) | 13(54.2) | 20(39.2) |  |
| III or IV, n (%) | 11(45.8) | 31(60.8) |  |
| NT-proBNP, ng/L | 648.7(123.4, 1588.3) | 757.7(209.0, 1979.0) | 0.370 |
| S_a_O_2_, % | 92.7±2.9 | 90.5±5.7 | 0.067 |
| 6MWD, m | 418.3±110.2 | 365.0±104.4 | 0.140 |
| Targeted therapy at baseline |  |  | **0.024** |
| None | 15(62.5) | 17(33.3) |  |
| Mono-therapy/ Combination | 9(37.5) | 34(66.7) |  |
| Echocardiography |  |  |  |
| LA, mm | 35.8±8.8 | 33.2±5.0 | 0.362 |
| LVED, mm | 43.5±6.1 | 40.4±5.1 | **0.023** |
| RVED, mm | 32.9±5.1 | 31.8±6.5 | 0.158 |
| RVED/LVED | 0.8±0.2 | 0.8±0.2 | 0.557 |
| EF, % | 64.8±6.1 | 65.0±5.7 | 0.927 |
| TRV, m/s | 4.1±0.6 | 4.4±0.7 | 0.097 |
| Hemodynamics |  |  |  |
| S_v_O_2_, % | 71.1±5.2 | 69.0±5.4 | 0.121 |
| mRAP, mmHg | 8.2±3.6 | 8.1±3.2 | 0.797 |
| sPAP, mmHg | 90.3±19.2 | 93.9±20.9 | 0.476 |
| dPAP, mmHg | 31±7.7 | 31.8±9.1 | 0.716 |
| mPAP, mmHg | 49.7±10.2 | 52.2±12.4 | 0.396 |
| PAWP, mmHg | 10.1±3.3 | 10.1±3.2 | 0.962 |
| Cardiac index, L /min/m^2^ | 2.9±0.8 | 2.9±0.8 | 0.875 |
| PVR, wood units | 9.2±4.4 | 10.5±4 | 0.198 |
| Cardiopulmonary exercise test |  |  |  |
| VO_2_@Peak, mL/min/kg | 14.4±3.1 | 11.6±3.0 | **0.001** |
| VO_2_@Peak, % predicted | 54.0±10.8 | 47.7±14.5 | 0.057 |
| VE/VCO_2_ slope | 45.2±9.2 | 50.9±9.7 | **0.020** |
| Pulmonary function test |  |  |  |
| FVC, % predicted | 73.1±5.6 | 74.6±6.9 | **0.008** |
| FEV_1_, % predicted | 96.4±19.5 | 85.3±14.4 | **0.033** |
| FEV_1_/FVC, % | 86.4±18 | 77.7±14.9 | 0.371 |
| DLCO, % predicted | 79.5±6.2 | 56.8±9.2 | **<0.001** |
| DLCO/VA, % predicted | 94±13.8 | 74±11.8 | **<0.001** |

Data are presented as mean ± standard deviation, median (range) or number (percentage). BMI, Body mass index; BPA, Balloon pulmonary angioplasty; DLCO, Diffusing capacity for carbon monoxide; dPAP, Diastolic pulmonary arterial pressure; EF, Ejection fraction; LA, Left atrium dimension; LVED, Left ventricular end-diastolic diameter; mPAP, Mean pulmonary arterial pressure; mRAP, Mean right atrial pressure; NT-proBNP, N-terminal pro-brain natriuretic peptide; PAWP, Pulmonary arterial wedge pressure; PVR, Pulmonary vascular resistance; RVED, Right ventricular end-diastolic diameter; 6MWD, 6-min walk distance; S_a_O_2_, Arterial oxygen saturation; sPAP, Systolic pulmonary arterial pressure; S_V_O_2_, Mixed venous oxygen saturation; TRV, Tricuspid regurgitation velocity; VA, Alveolar ventilation; VE/VCO_2_ slope, Minute ventilation/ carbon dioxide output slope; VO_2_@Peak, Peak oxygen consumption; WHO FC, World Health Organization functional class. Bold values mean their *P* value < 0.050.

Table S3 Clinical Features of Patients with DLCO%<70% and DLCO%≥ 70% at Follow-up

| Variables | DLCO≥ 70% (n=24) | DLCO<70% (n=51) | *P* value |
| --- | --- | --- | --- |
| Number of BPA sessions | 2.3±1.3 | 2.2±1.3 | 0.740 |
| Number of dilated subsegmental pulmonary vessels | 16.1±9.3 | 14.1±9.5 | 0.239 |
| Time interval*, weeks | 40.9±27.1 | 36.7±26.9 | 0.578 |
| BPA responder, % | 19(79.2) | 26(51) | **0.020** |
| WHO FC |  |  | 1.000 |
| I or II, n (%) | 21(87.5) | 45(88.2) |  |
| III or IV, n (%) | 3(12.5) | 6(11.8) |  |
| NT-proBNP, ng/L | 91.6(44.0, 410.7) | 212.0(77.2, 709.0) | 0.071 |
| S_a_O_2_, % | 94.0±2.4 | 92.5±5 | 0.053 |
| 6MWD, m | 490.0±104.6 | 410.1±65.8 | **0.001** |
| Echocardiography |  |  |  |
| LA, mm | 35.7±7.8 | 34.3±6.8 | 0.439 |
| LVED, mm | 46.2±5.3 | 42.9±3.9 | **0.003** |
| RVED, mm | 29.0±4.5 | 28.8±6.5 | 0.487 |
| RVED/LVED | 0.6±0.1 | 0.7±0.2 | 0.291 |
| EF, % | 65.8±6.4 | 64.4±5.4 | 0.529 |
| TRV, m/s | 3.7±0.6 | 4.0±0.7 | 0.088 |
| Hemodynamics |  |  |  |
| S_v_O_2_, % | 72.7±4.7 | 71.5±5.2 | 0.338 |
| mRAP, mmHg | 6.5±3.2 | 7.1±3.1 | 0.445 |
| sPAP, mmHg | 63.5±18.7 | 73.3±24.5 | 0.086 |
| dPAP, mmHg | 22.8±5.9 | 25.2±7.5 | 0.237 |
| mPAP, mmHg | 36.0±8.2 | 41.0±12.0 | 0.096 |
| PAWP, mmHg | 10.3±4.1 | 10.3±3.3 | 0.813 |
| Cardiac index, L /min/m^2^ | 3.2±0.5 | 3.4±1.0 | 0.809 |
| PVR, wood units | 5.2±2.5 | 6.9±3.1 | **0.027** |
| Cardiopulmonary exercise test |  |  |  |
| VO_2_@Peak, mL/min/kg | 16.6±3.6 | 13.4±3.2 | **<0.001** |
| VO_2_@Peak, % predicted | 63.5±15.5 | 55.6±15.5 | 0.051 |
| VE/VCO_2_ slope | 38.5±8.3 | 44.6±7.5 | **0.003** |
| Pulmonary function test |  |  |  |
| FVC, % predicted | 95.2±16.5 | 88±16.1 | 0.091 |
| FEV_1_, % predicted | 86.8±16.5 | 79.3±17.5 | 0.100 |
| FEV_1_/FVC, % | 74.2±5.3 | 73.7±6.8 | 0.783 |
| DLCO, % predicted | 78.4±10 | 59.9±11 | **<0.001** |
| DLCO/VA, % predicted | 90.6±13.9 | 74.4±12 | **<0.001** |

Data are presented as mean ± standard deviation, median (range) or number (percentage). BPA, Balloon pulmonary angioplasty; DLCO, Diffusing capacity for carbon monoxide; dPAP, Diastolic pulmonary arterial pressure; EF, Ejection fraction; LA, Left atrium dimension; LVED, Left ventricular end-diastolic diameter; mPAP, Mean pulmonary arterial pressure; mRAP, Mean right atrial pressure; NT-proBNP, N-terminal pro-brain natriuretic peptide; PAWP, Pulmonary arterial wedge pressure; PVR, Pulmonary vascular resistance; RVED, Right ventricular end-diastolic diameter; 6MWD, 6-min walk distance; S_a_O_2_, Arterial oxygen saturation; sPAP, Systolic pulmonary arterial pressure; S_V_O_2_, Mixed venous oxygen saturation; TRV, Tricuspid regurgitation velocity; VA, Alveolar ventilation; VE/VCO_2_ slope, Minute ventilation/ carbon dioxide output slope; VO_2_@Peak, Peak oxygen consumption; WHO FC, World Health Organization functional class. Bold values mean their *P* value < 0.050. *Time interval between baseline and follow-up.

Table S4 Clinical Features of Patients with ΔDLCO≤6% and ΔDLCO>6% at Baseline.

| Variables | ΔDLCO≤6% (n=64) | ΔDLCO>6% (n=11) | *P* value |
| --- | --- | --- | --- |
| Age, years | 56.1±12.2 | 65.1±6.5 | **0.020** |
| Female, n (%) | 36(56.3) | 4(36.4) | 0.328 |
| BMI, kg/m^2^ | 24.0±3.2 | 23.9±3.8 | 0.899 |
| WHO FC |  |  | 0.746 |
| I or II, n (%) | 29(45.3) | 4(36.4) |  |
| III or IV, n (%) | 35(54.7) | 7(63.6) |  |
| NT-proBNP, ng/L | 740.4(177.5, 1710.0) | 638.8(201.0, 2528.0) | 0.952 |
| S_a_O_2_, % | 91.1±5.4 | 91.9±2.0 | 0.928 |
| 6MWD, m | 380.3±108.8 | 396.1±111.6 | 0.685 |
| Targeted therapy at baseline |  |  | **0.019** |
| None | 31(48.4) | 1(9.1) |  |
| Mono-therapy/ Combination | 33(51.6) | 10(90.9) |  |
| Echocardiography |  |  |  |
| LA, mm | 33.7±6.6 | 36.2±6.2 | 0.141 |
| LVED, mm | 41.2±5.4 | 43±6.7 | 0.315 |
| RVED, mm | 32.2±5.9 | 32.4±7.3 | 0.910 |
| RVED/LVED | 0.8±0.2 | 0.8±0.3 | 0.771 |
| EF, % | 64.8±5.8 | 66.0±5.9 | 0.289 |
| TRV, m/s | 4.3±0.7 | 4.4±0.6 | 0.750 |
| Hemodynamics |  |  |  |
| S_v_O_2_, % | 69.6±5.1 | 69.8±7.1 | 0.908 |
| mRAP, mmHg | 8.0±3.4 | 8.9±2.5 | 0.249 |
| sPAP, mmHg | 92.3±21 | 95±16.3 | 0.688 |
| dPAP, mmHg | 31.5±8.6 | 32.3±8.8 | 0.621 |
| mPAP, mmHg | 51.2±11.9 | 52.5±11.4 | 0.410 |
| PAWP, mmHg | 10.0±3.1 | 10.5±4.0 | 0.875 |
| Cardiac index, L /min/m^2^ | 2.9±0.8 | 3.1±0.7 | 0.286 |
| PVR, wood units | 10.1±4.1 | 10.1±4.5 | 0.960 |
| Cardiopulmonary exercise test |  |  |  |
| VO_2_@Peak, mL/min/kg | 12.6±3.2 | 11.8±3.9 | 0.287 |
| VO_2_@Peak, % predicted | 50.1±13.6 | 47.6±14.4 | 0.579 |
| VE/VCO_2_ slope | 48.6±9.3 | 51.4±12.7 | 0.398 |
| Pulmonary function test |  |  |  |
| FVC, % predicted | 89.7±17.2 | 83.7±14.3 | 0.282 |
| FEV_1_, % predicted | 80.9±16.1 | 77.5±18.3 | 0.519 |
| FEV_1_/FVC, % | 74.3±6.7 | 73.1±5.6 | 0.580 |
| DLCO, % predicted | 65.4±12.7 | 56.1±15.8 | **0.034** |
| DLCO/VA, % predicted | 81.0±15.5 | 76.8±16.1 | 0.412 |

Data are presented as mean ± standard deviation, median (range) or number (percentage). BMI, Body mass index; BPA, Balloon pulmonary angioplasty; DLCO, Diffusing capacity for carbon monoxide; ΔDLCO, (DLCO after first BPA session- DLCO before first BPA session)/ DLCO before first BPA session; dPAP, Diastolic pulmonary arterial pressure; EF, Ejection fraction; LA, Left atrium dimension; LVED, Left ventricular end-diastolic diameter; mPAP, Mean pulmonary arterial pressure; mRAP, Mean right atrial pressure; NT-proBNP, N-terminal pro-brain natriuretic peptide; PAWP, Pulmonary arterial wedge pressure; PVR, Pulmonary vascular resistance; RVED, Right ventricular end-diastolic diameter; 6MWD, 6-min walk distance; S_a_O_2_, Arterial oxygen saturation; sPAP, Systolic pulmonary arterial pressure; S_V_O_2_, Mixed venous oxygen saturation; TRV, Tricuspid regurgitation velocity; VA, Alveolar ventilation; VE/VCO_2_ slope, Minute ventilation/ carbon dioxide output slope; VO_2_@Peak, Peak oxygen consumption; WHO FC, World Health Organization functional class. Bold values mean their *P* value < 0.050.

Table S5 Clinical Features of Patients with ΔDLCO≤6% and ΔDLCO>6% at Follow-up

| Variables | ΔDLCO≤6% (n=64) | ΔDLCO>6% (n=11) | *P* value |
| --- | --- | --- | --- |
| Number of BPA sessions | 2.3±1.3 | 2±1.2 | 0.542 |
| Number of dilated subsegmental pulmonary vessels | 14.9±9.6 | 13.7±8.6 | 0.759 |
| Time interval*, weeks | 39.6±27.4 | 29.2±22.5 | 0.385 |
| BPA responder, % | 42(65.6) | 3(27.3) | **0.022** |
| WHO FC |  |  | 1.000 |
| I or II, n (%) | 56(87.5) | 10(90.9) |  |
| III or IV, n (%) | 8(12.5) | 1(9.1) |  |
| NT-proBNP, ng/L | 150.3(54.3, 685.1) | 157.7(67.3, 265) | 0.964 |
| S_a_O_2_, % | 93.0±4.7 | 93.0±1.7 | 0.350 |
| 6MWD, m | 439.6±88.7 | 422.0±90.8 | 0.590 |
| Echocardiography |  |  |  |
| LA, mm | 34.1±6 | 38.2±11.5 | 0.154 |
| LVED, mm | 43.8±4.5 | 44.6±5.4 | 0.583 |
| RVED, mm | 28.6±5.9 | 30.5±5.8 | 0.286 |
| RVED/LVED | 0.7±0.2 | 0.7±0.2 | 0.373 |
| EF, % | 65.1±6.1 | 63.5±2.8 | 0.269 |
| TRV, m/s | 3.8±0.7 | 4.1±0.7 | 0.272 |
| Hemodynamics |  |  |  |
| S_v_O_2_, % | 72.1±5 | 70.4±5.4 | 0.292 |
| mRAP, mmHg | 6.9±3.2 | 7.3±2.9 | 0.438 |
| sPAP, mmHg | 68.2±23.5 | 81.8±18.1 | 0.070 |
| dPAP, mmHg | 23.8±7 | 27.9±6.8 | 0.076 |
| mPAP, mmHg | 38.5±11.1 | 44.8±9.6 | **0.033** |
| PAWP, mmHg | 10.2±3.8 | 11.1±2.0 | 0.448 |
| Cardiac index, L /min/m^2^ | 3.2±0.7 | 3.6±1.5 | 0.969 |
| PVR, wood units | 6.1±3 | 7.7±2.6 | **0.043** |
| Cardiopulmonary exercise test |  |  |  |
| VO_2_@Peak, mL/min/kg | 14.6±3.3 | 13.3±4.9 | 0.299 |
| VO_2_@Peak, % predicted | 58.8±15.3 | 53.5±19.3 | 0.332 |
| VE/VCO_2_ slope | 42.3±8.2 | 44.9±8.2 | 0.359 |
| Pulmonary function test |  |  |  |
| FVC, % predicted | 91.6±15.9 | 81.7±17.5 | 0.660 |
| FEV_1_, % predicted | 82.8±16.1 | 74.6±23 | 0.157 |
| FEV_1_/FVC, % | 74.3±6.1 | 71.4±7.6 | 0.160 |
| DLCO, % predicted | 66±12.8 | 63.2±18.2 | 0.533 |
| DLCO/VA, % predicted | 79.2±14.5 | 80.2±15.9 | 0.841 |

Data are presented as mean ± standard deviation, median (range) or number (percentage). BPA, Balloon pulmonary angioplasty; DLCO, Diffusing capacity for carbon monoxide; ΔDLCO, (diffusing capacity for carbon monoxide after first BPA session- diffusing capacity for carbon monoxide before first BPA session)/ diffusing capacity for carbon monoxide before first BPA session; dPAP, Diastolic pulmonary arterial pressure; EF, Ejection fraction; LA, Left atrium dimension; LVED, Left ventricular end-diastolic diameter; mPAP, Mean pulmonary arterial pressure; mRAP, Mean right atrial pressure; NT-proBNP, N-terminal pro-brain natriuretic peptide; PAWP, Pulmonary arterial wedge pressure; PVR, Pulmonary vascular resistance; RVED, Right ventricular end-diastolic diameter; 6MWD, 6-min walk distance; S_a_O_2_, Arterial oxygen saturation; sPAP, Systolic pulmonary arterial pressure; S_V_O_2_, Mixed venous oxygen saturation; TRV, Tricuspid regurgitation velocity; VA, Alveolar ventilation; VE/VCO_2_ slope, Minute ventilation/ carbon dioxide output slope; VO_2_@Peak, Peak oxygen consumption; WHO FC, World Health Organization functional class. Bold values mean their *P* value < 0.050. *Time interval between baseline and follow-up.

Table S6 Clinical Features of BPA Responders and Non-responders at Baseline (Sensitivity Analysis for baseline DLCO<70%)

| Variables | Responder (n=54) | Non-responder (n=38) | *P* value |
| --- | --- | --- | --- |
| Age, years | 58.2±11.9 | 58.8±11.0 | 0.803 |
| Female, n (%) | 28(51.9) | 19(50.0) | 0.861 |
| WHO FC |  |  | 0.078 |
| I or II, n (%) | 27(50.0) | 12(31.6) |  |
| III or IV, n (%) | 27(50.0) | 26(68.4) |  |
| NT-proBNP, ng/L | 577.8(107.5, 1532.8) | 906.6(326.0, 2324.0) | 0.064 |
| S_a_O_2_, % | 91.9±2.7 | 91.1±3.7 | 0.198 |
| 6MWD, m | 381.6±95.1 | 357.0±124.6 | 0.300 |
| Targeted therapy at baseline |  |  | 0.183 |
| None | 26(48.1) | 13(34.2) |  |
| Mono-therapy/ Combination | 28(51.9) | 25(65.8) |  |
| Echocardiography |  |  |  |
| LA, mm | 33.7±7.0 | 34.7±5.4 | 0.464 |
| LVED, mm | 41.1±5.9 | 41.9±5.0 | 0.296 |
| RVED, mm | 31.7±5.9 | 32.7±6.8 | 0.453 |
| RVED/LVED | 0.8±0.2 | 0.8±0.2 | 0.856 |
| EF, % | 65.0±5.8 | 64.7±4.9 | 0.958 |
| TRV, m/s | 4.3±0.7 | 4.4±0.6 | 0.496 |
| Hemodynamics |  |  |  |
| S_v_O_2_, % | 69.8±5.1 | 68.7±5.5 | 0.360 |
| mRAP, mmHg | 7.7±3.2 | 8.1±3.8 | 0.615 |
| sPAP, mmHg | 91.6±21.7 | 89.8±22.7 | 0.695 |
| dPAP, mmHg | 30.4±8.8 | 37.8±35.0 | 0.141 |
| mPAP, mmHg | 50.4±12.1 | 51.6±10.8 | 0.626 |
| PAWP, mmHg | 9.4±3.1 | 10.8±3.3 | 0.068 |
| Cardiac index, L /min/m^2^ | 3.0±0.8 | 2.7±0.7 | 0.142 |
| PVR, wood units | 10.1±4.4 | 9.8±3.5 | 0.709 |
| Cardiopulmonary exercise test |  |  |  |
| VO_2_@Peak, mL/min/kg | 12.8±3.9 | 12.2±2.7 | 0.381 |
| VO_2_@Peak, % predicted | 51.0±14.6 | 49.0±12.1 | 0.494 |
| VE/VCO_2_ slope | 49.0±9.6 | 48.9±9.0 | 0.943 |
| Pulmonary function test |  |  |  |
| FVC, % predicted | 91.4±14.3 | 85.1±19.5 | 0.083 |
| FEV_1_, % predicted | 83.6±15.8 | 76.3±17.6 | **0.042** |
| FEV_1_/FVC, % | 74.6±6.6 | 73.0±7.2 | 0.276 |
| DLCO, % predicted | 66.6±13.5 | 59.0±12.7 | **0.008** |
| DLCO/VA, % predicted | 81.7±16.5 | 75.9±12.5 | 0.130 |
| Number of BPA sessions | 2.6±1.5 | 1.9±1.0 | **0.030** |
| Number of dilated subsegmental pulmonary vessels | 16.7±10.5 | 12.1±6.7 | **0.049** |
| Time interval*, weeks | 39.0(22.8-69.3) | 29.5(13.5-49.8) | 0.118 |

Data are presented as mean ± standard deviation, median (range) or number (percentage). BPA, Balloon pulmonary angioplasty; DLCO, Diffusing capacity for carbon monoxide; dPAP, Diastolic pulmonary arterial pressure; EF, Ejection fraction; FVC, Forced vital capacity; FEV_1_, Forced expiratory volume in one second; LA, Left atrium dimension; LVED, Left ventricular end-diastolic diameter; mPAP, Mean pulmonary arterial pressure; mRAP, Mean right atrial pressure; NT-proBNP, N-terminal pro-brain natriuretic peptide; PAWP, Pulmonary arterial wedge pressure; PVR, Pulmonary vascular resistance; RVED, Right ventricular end-diastolic diameter; 6MWD, 6-min walk distance; S_a_O_2_, Arterial oxygen saturation; sPAP, Systolic pulmonary arterial pressure; S_V_O_2_, Mixed venous oxygen saturation; TRV, Tricuspid regurgitation velocity; VA, Alveolar ventilation; VE/VCO_2_ slope, Minute ventilation/ carbon dioxide output slope; VO_2_@Peak, Peak oxygen consumption; WHO FC, World Health Organization functional class. Bold values mean their *P* value < 0.050. *Time interval between baseline and follow-up.

Table S7 Clinical Features of BPA Responders and Non-responders at Follow-up (Sensitivity Analysis for baseline DLCO<70%)

| Variables | Responder (n=54) | Non-responder (n=38) | *P* value |
| --- | --- | --- | --- |
| WHO FC |  |  | 0.058 |
| I or II, n (%) | 49(90.7) | 29(76.3) |  |
| III or IV, n (%) | 5(9.3) | 9(23.7) |  |
| NT-proBNP, ng/L | 105.5(59.8, 243.7) | 300.6(98.5, 788.3) | **0.006** |
| S_a_O_2_, % | 93.7±2.6 | 91.3±6.1 | **0.004** |
| 6MWD, m | 437.6±85.1 | 428.1±89.0 | 0.669 |
| Echocardiography |  |  |  |
| LA, mm | 34.4±5.9 | 35.1±7.8 | 0.632 |
| LVED, mm | 44.6±4.7 | 43.4±4.2 | 0.218 |
| RVED, mm | 28.3±5.1 | 29.9±6.4 | 0.228 |
| RVED/LVED | 0.6±0.1 | 0.7±0.2 | 0.107 |
| EF, % | 66.3±4.9 | 62.9±5.6 | **0.008** |
| TRV, m/s | 3.6±0.7 | 4.2±0.6 | **0.001** |
| Hemodynamics |  |  |  |
| S_v_O_2_, % | 72.6±4.7 | 70.0±5.3 | **0.014** |
| mRAP, mmHg | 6.6±3.1 | 7.1±3.2 | 0.473 |
| sPAP, mmHg | 62.0±20.9 | 82.8±20.6 | **<0.001** |
| dPAP, mmHg | 21.5±5.4 | 28.8±7.1 | **<0.001** |
| mPAP, mmHg | 34.8±9.1 | 46.1±10.2 | **<0.001** |
| PAWP, mmHg | 10.5±3.6 | 9.9±3.5 | 0.470 |
| Cardiac index, L /min/m^2^ | 3.3±0.6 | 3.2±1.1 | 0.730 |
| PVR, wood units | 5.1±2.3 | 9.0±3.3 | **<0.001** |
| Cardiopulmonary exercise test |  |  |  |
| VO_2_@Peak, mL/min/kg | 15.0±3.8 | 13.5±3.6 | 0.077 |
| VO_2_@Peak, % predicted | 61.0±15.2 | 53.4±15.3 | **0.027** |
| VE/VCO_2_ slope | 41.3±7.8 | 44.9±7.3 | **0.033** |
| Pulmonary function test |  |  |  |
| FVC, % predicted | 93.2±15.1 | 86.2±17.7 | 0.054 |
| FEV_1_, % predicted | 85.7±16.7 | 76.4±17.9 | **0.016** |
| FEV_1_/FVC, % | 74.9±5.9 | 72.1±7.3 | **0.038** |
| DLCO, % predicted | 67.1±15.4 | 62.8±12.6 | 0.179 |
| DLCO/VA, % predicted | 78.1±15.7 | 76.0±12.3 | 0.693 |

Data are presented as mean ± standard deviation, median (range) or number (percentage). BPA, Balloon pulmonary angioplasty; DLCO, Diffusing capacity for carbon monoxide; dPAP, Diastolic pulmonary arterial pressure; EF, Ejection fraction; FVC, Forced vital capacity; FEV_1_, Forced expiratory volume in one second; LA, Left atrium dimension; LVED, Left ventricular end-diastolic diameter; mPAP, Mean pulmonary arterial pressure; mRAP, Mean right atrial pressure; NT-proBNP, N-terminal pro-brain natriuretic peptide; PAWP, Pulmonary arterial wedge pressure; PVR, Pulmonary vascular resistance; RVED, Right ventricular end-diastolic diameter; 6MWD, 6-min walk distance; S_a_O_2_, Arterial oxygen saturation; sPAP, Systolic pulmonary arterial pressure; S_V_O_2_, Mixed venous oxygen saturation; TRV, Tricuspid regurgitation velocity; VA, Alveolar ventilation; VE/VCO_2_ slope, Minute ventilation/ carbon dioxide output slope; VO_2_@Peak, Peak oxygen consumption; WHO FC, World Health Organization functional class. Bold values mean their *P* value < 0.050.

# Table S8 Clinical Features of Patients with DLCO<70% and DLCO≥70% at Baseline (Sensitivity Analysis for baseline DLCO<70%)

| Variables | DLCO%<70% (n=65) | DLCO%≥70% (n=27) | *P* value |
| --- | --- | --- | --- |
| WHO FC |  |  | 0.237 |
| I or II, n (%) | 25(38.5) | 14(51.9) |  |
| III or IV, n (%) | 40(61.5) | 13(48.1) |  |
| NT-proBNP, ng/L | 818.2(210.85, 1759) | 638.8(106.6, 1139.1) | 0.232 |
| S_a_O_2_, % | 91.29±3.17 | 92.24±2.95 | 0.182 |
| 6MWD, m | 352.03±103 | 417.92±107 | **0.030** |
| Targeted therapy at baseline |  |  | **0.009** |
| None | 21(32.3) | 18(66.7) |  |
| Mono-therapy | 35(53.8) | 8(29.6) |  |
| Combination therapy | 9(13.8) | 1(3.7) |  |
| Echocardiography |  |  |  |
| LA, mm | 33.45±5.18 | 35.59±8.48 | 0.447 |
| LVED, mm | 40.74±5.06 | 43.11±6.24 | 0.060 |
| RVED, mm | 32.02±6.64 | 32.3±5.27 | 0.369 |
| RVED/LVED | 0.81±0.23 | 0.77±0.18 | 0.754 |
| EF, % | 65.02±5.19 | 64.56±5.98 | 0.477 |
| TRV, m/s | 4.38±0.63 | 4.17±0.65 | 0.153 |
| Hemodynamics |  |  |  |
| S_v_O_2_, % | 68.62±5.29 | 71.02±4.93 | **0.047** |
| mRAP, mmHg | 7.72±3.45 | 8.11±3.4 | 0.509 |
| sPAP, mmHg | 91.8±22.63 | 88.63±20.64 | 0.532 |
| dPAP, mmHg | 34.43±27.66 | 31.04±7.56 | 0.935 |
| mPAP, mmHg | 51.55±11.99 | 49.15±10.41 | 0.366 |
| PAWP, mmHg | 9.93±3.29 | 9.93±3.15 | 0.996 |
| Cardiac index, L/min/m^2^ | 2.88±0.82 | 2.87±0.76 | 0.935 |
| PVR, wood units | 10.33±3.98 | 9.11±4.23 | 0.192 |
| Cardiopulmonary exercise test |  |  |  |
| VO_2_@Peak, mL/min/kg | 11.69±2.93 | 14.65±3.69 | **<0.001** |
| VO_2_@Peak, % predicted | 47.92±13.59 | 55.56±12.32 | **0.019** |
| VE/VCO_2_ slope | 50.65±9.04 | 45.03±8.86 | **0.014** |

Data are presented as mean ± standard deviation, median (range) or number (percentage). BPA, Balloon pulmonary angioplasty; DLCO, Diffusing capacity for carbon monoxide; dPAP, Diastolic pulmonary arterial pressure; EF, Ejection fraction; FVC, Forced vital capacity; FEV_1_, Forced expiratory volume in one second; LA, Left atrium dimension; LVED, Left ventricular end-diastolic diameter; mPAP, Mean pulmonary arterial pressure; mRAP, Mean right atrial pressure; NT-proBNP, N-terminal pro-brain natriuretic peptide; PAWP, Pulmonary arterial wedge pressure; PVR, Pulmonary vascular resistance; RVED, Right ventricular end-diastolic diameter; 6MWD, 6-min walk distance; S_a_O_2_, Arterial oxygen saturation; sPAP, Systolic pulmonary arterial pressure; S_V_O_2_, Mixed venous oxygen saturation; TRV, Tricuspid regurgitation velocity; VE/VCO_2_ slope, Minute ventilation/ carbon dioxide output slope; VO_2_@Peak, Peak oxygen consumption; WHO FC, World Health Organization functional class. Bold values mean their *P* value < 0.050.

# Table S9 Clinical Features of Patients with DLCO<70% and DLCO≥70% at Follow-up (Sensitivity Analysis for baseline DLCO<70%)

| Variables | DLCO%<70% (n=65) | DLCO%≥70%  (n=27) | *P* value |
| --- | --- | --- | --- |
| WHO FC |  |  | 0.463 |
| I or II, n (%) | 54(83.1) | 24(88.9) |  |
| III or IV, n (%) | 11(16.9) | 3(11.1) |  |
| NT-proBNP, ng/L | 212(79.7, 661.1) | 89.2(43.8, 258) | **0.015** |
| S_a_O_2_, % | 92.13±5.07 | 94.06±2.45 | **0.017** |
| 6MWD, m | 408.09±65.45 | 490.05±100.27 | **<0.001** |
| Echocardiography |  |  |  |
| LA, mm | 34.45±6.44 | 35.41±7.43 | 0.618 |
| LVED, mm | 43.35±3.99 | 45.85±5.22 | 0.015 |
| RVED, mm | 29.05±6.19 | 28.81±4.39 | 0.712 |
| RVED/LVED | 0.68±0.17 | 0.64±0.12 | 0.270 |
| EF, % | 64.57±5.15 | 65.67±5.99 | 0.585 |
| TRV, m/s | 3.95±0.74 | 3.62±0.65 | 0.050 |
| Hemodynamics |  |  |  |
| S_v_O_2_, % | 71.04±5.17 | 72.74±4.77 | 0.146 |
| mRAP, mmHg | 7±3.14 | 6.41±3.07 | 0.327 |
| sPAP, mmHg | 74.23±23.89 | 61.78±18.5 | **0.017** |
| dPAP, mmHg | 25.25±7.43 | 22.74±6.09 | 0.125 |
| mPAP, mmHg | 41.15±11.68 | 35.48±8.27 | **0.026** |
| PAWP, mmHg | 10.24±3.43 | 10.37±3.88 | 0.933 |
| Cardiac index, L/min/m^2^ | 3.28±0.92 | 3.21±0.49 | 0.626 |
| PVR, wood units | 7.25±3.48 | 5.21±2.48 | **0.006** |
| Cardiopulmonary exercise test |  |  |  |
| VO_2_@Peak, mL/min/kg | 13.34±3.25 | 16.92±3.74 | **<0.001** |
| VO_2_@Peak, % predicted | 54.93±14.7 | 65.28±15.54 | **0.005** |
| VE/VCO_2_ slope | 44.53±7.04 | 38.43±7.97 | **0.001** |
| Number of BPA sessions | 2.23±1.27 | 2.22±1.42 | 0.897 |
| BPA responder, % | 33(50.8) | 21(77.8) | **0.017** |

Data are presented as mean ± standard deviation, median (range) or number (percentage). BPA, Balloon pulmonary angioplasty; DLCO, Diffusing capacity for carbon monoxide; dPAP, Diastolic pulmonary arterial pressure; EF, Ejection fraction; LA, Left atrium dimension; LVED, Left ventricular end-diastolic diameter; mPAP, Mean pulmonary arterial pressure; mRAP, Mean right atrial pressure; NT-proBNP, N-terminal pro-brain natriuretic peptide; PAWP, Pulmonary arterial wedge pressure; PVR, Pulmonary vascular resistance; RVED, Right ventricular end-diastolic diameter; 6MWD, 6-min walk distance; S_a_O_2_, Arterial oxygen saturation; sPAP, Systolic pulmonary arterial pressure; S_V_O_2_, Mixed venous oxygen saturation; TRV, Tricuspid regurgitation velocity; VE/VCO_2_ slope, Minute ventilation/ carbon dioxide output slope; VO_2_@Peak, Peak oxygen consumption; WHO FC, World Health Organization functional class. Bold values mean their *P* value < 0.050.

Table S10 Univariate Logistic Regression Analyses for Unfavorable Hemodynamic Response to BPA (Sensitivity Analysis for Baseline DLCO<70%)

| Variable | OR | 95%CI | *P*-value |
| --- | --- | --- | --- |
| Age | 1.005 | 0.968-1.042 | 0.800 |
| Female | 0.929 | 0.405-2.130 | 0.861 |
| WHO FC | 2.167 | 0.910-5.158 | 0.081 |
| Ln(NT-proBNP) | 1.334 | 0.981-1.815 | 0.066 |
| 6MWD | 0.998 | 0.994-1.002 | 0.298 |
| None/ Medicine treatment | 1.786 | 0.758-4.206 | 0.185 |
| RVED/LVED | 1.200 | 0.171-8.402 | 0.854 |
| EF | 0.992 | 0.918-1.071 | 0.830 |
| TRV | 1.261 | 0.651-2.443 | 0.492 |
| S_v_O_2_ | 0.963 | 0.889-1.043 | 0.356 |
| mRAP | 1.032 | 0.914-1.166 | 0.611 |
| dPAP | 1.028 | 0.98-1.078 | 0.263 |
| mPAP | 1.009 | 0.973-1.046 | 0.622 |
| PAWP | 1.150 | 0.999-1.323 | 0.051 |
| Cardiac index | 0.600 | 0.334-1.081 | 0.089 |
| PVR | 0.980 | 0.883-1.088 | 0.706 |
| FEV_1_/FVC | 0.966 | 0.909-1.028 | 0.274 |
| DLCO | 0.957 | 0.924-0.99 | **0.011** |
| DLCO<70% | 3.394 | 1.212-9.501 | **0.020** |
| DLCO/VA %predicted | 0.974 | 0.946-1.002 | 0.072 |
| VO_2_@Peak | 0.949 | 0.837-1.076 | 0.411 |
| VE/VCO_2_ slope | 0.998 | 0.954-1.045 | 0.942 |
| Number of BPA sessions | 0.636 | 0.441-0.918 | **0.016** |
| Number of dilated subsegmental pulmonary vessels | 0.941 | 0.894-0.992 | **0.024** |
| Time interval* | 0.985 | 0.970-1.001 | 0.069 |

Baseline variable were included in analysis. BPA, Balloon pulmonary angioplasty; dPAP, Diastolic pulmonary arterial pressure; DLCO, Diffusing capacity for carbon monoxide; EF, Ejection fraction; FVC, Forced vital capacity; FEV_1_, Forced expiratory volume in one second; LVED, Left ventricular end-diastolic diameter; mPAP, Mean pulmonary arterial pressure; mRAP, Mean right atrial pressure; NT-proBNP, N-terminal pro-brain natriuretic peptide; PAWP, Pulmonary arterial wedge pressure; PVR, Pulmonary vascular resistance; RVED, Right ventricular end-diastolic diameter; 6MWD, 6-min walk distance; S_v_O_2_, Mixed venous oxygen saturation; TRV, Tricuspid regurgitation velocity; VA, Alveolar ventilation; VE/VCO_2_ slope, Minute ventilation/ carbon dioxide output slope; VO_2_@Peak, Peak oxygen consumption; WHO FC, World Health Organization functional class. Bold values mean their *P* value < 0.050. *Time interval between baseline and follow-up.

Table S11 Multivariate Logistic Regression Analyses for Unfavorable Hemodynamic Response to BPA (Sensitivity Analysis for Baseline DLCO<70%)

| Model | Variable | OR | 95%CI | *P*-value |
| --- | --- | --- | --- | --- |
| 1 | DLCO<70% | 3.337 | 1.157-9.628 | **0.026** |
|  | Number of BPA sessions | 0.637 | 0.436-0.932 | **0.020** |
| 2 | DLCO<70% | 3.119 | 1.069-9.104 | **0.037** |
|  | Number of BPA sessions | 0.643 | 0.439-0.942 | **0.023** |
|  | WHO FC | 1.936 | 0.77-4.867 | 0.160 |
| 3 | DLCO<70% | 3.042 | 1.031-8.979 | **0.044** |
|  | Number of BPA sessions | 0.618 | 0.418-0.912 | **0.015** |
|  | Ln(NT-proBNP) | 1.349 | 0.965-1.885 | 0.080 |
| 4 | DLCO<70% | 3.221 | 1.098-9.452 | **0.033** |
|  | Number of BPA sessions | 0.640 | 0.438-0.934 | **0.021** |
|  | S_v_O_2_ | 0.984 | 0.903-1.072 | 0.719 |
| 5 | DLCO<70% | 3.220 | 1.111-9.337 | **0.031** |
|  | Number of BPA sessions | 0.628 | 0.428-0.922 | **0.018** |
|  | mPAP | 1.012 | 0.973-1.053 | 0.553 |
| 6 | DLCO<70% | 3.627 | 1.191-11.043 | **0.023** |
|  | Number of BPA sessions | 0.597 | 0.396-0.902 | **0.014** |
|  | PAWP | 1.154 | 0.991-1.343 | 0.065 |
| 7 | DLCO<70% | 3.716 | 1.179-11.714 | **0.025** |
|  | Number of BPA sessions | 0.654 | 0.43-0.994 | **0.047** |
|  | Cardiac index | 0.607 | 0.323-1.143 | 0.122 |

BPA, Balloon pulmonary angioplasty; DLCO, Diffusing capacity for carbon monoxide; mPAP, Mean pulmonary arterial pressure; NT-proBNP, N-terminal pro-brain natriuretic peptide; PAWP, Pulmonary arterial wedge pressure; PVR, Pulmonary vascular resistance; S_v_O_2_, Mixed venous oxygen saturation; WHO FC, World Health Organization functional class. Bold values mean their *P* value < 0.050.

Table S12 Clinical Features of Included and Excluded Patients at Baseline

| Variables | Excluded patients (n=51) | Included patients (n=75) | *P* value |
| --- | --- | --- | --- |
| Age, years | 62.3±8.1 | 57.5±11.9 | **0.035** |
| Female, n (%) | 28(54.9) | 40(53.3) | 0.862 |
| WHO FC |  |  | **0.010** |
| I or II, n (%) | 11(21.6) | 33(44.0) |  |
| III or IV, n (%) | 40(78.4) | 42(56.0) |  |
| NT-proBNP, ng/L | 1139.1(206.5, 2013.0) | 719.0(189.1, 1816.0) | 0.282 |
| S_a_O_2_, % | 91.3±2.9 | 91.7±3.2 | 0.326 |
| 6MWD, m | 342.3±108.8 | 382.3±108.5 | 0.065 |
| Targeted therapy at baseline |  |  | 0.868 |
| None | 21(41.2) | 32(42.7) |  |
| Mono-therapy/ Combination | 30(58.8) | 43(57.3) |  |
| Echocardiography |  |  |  |
| LA, mm | 34.7±5.9 | 34.1±6.5 | 0.245 |
| LVED, mm | 40.4±5.9 | 41.4±5.6 | 0.301 |
| RVED, mm | 33.5±6.7 | 32.2±6.1 | 0.293 |
| EF, % | 66.5±6 | 64.9±5.8 | 0.160 |
| TRV, m/s | 4.3±0.6 | 4.3±0.6 | 0.975 |
| Hemodynamics |  |  |  |
| S_v_O_2_, % | 67.9±7.2 | 69.6±5.4 | 0.114 |
| mRAP, mmHg | 8±4.3 | 8.1±3.3 | 0.721 |
| sPAP, mmHg | 85.9±25 | 92.7±20.3 | 0.096 |
| dPAP, mmHg | 34.1±30.9 | 31.6±8.6 | 0.557 |
| mPAP, mmHg | 48.7±11.5 | 51.4±11.8 | 0.306 |
| PAWP, mmHg | 10±3.3 | 10.1±3.2 | 0.867 |
| Cardiac index, L/min/m^2^ | 2.7±0.8 | 2.9±0.8 | 0.245 |
| PVR, wood units | 10.4±5.1 | 10.1±4.2 | 0.737 |
| Cardiopulmonary exercise test |  |  |  |
| VO_2_@Peak, mL/min/kg | 12.3±4.1 | 12.5±3.3 | 0.837 |
| VO_2_@Peak, % predicted | 52.3±15.3 | 49.8±13.7 | 0.393 |
| VE/VCO_2_ slope | 47.2±8.7 | 49±9.9 | 0.361 |

Data are presented as mean ± standard deviation, median (range) or number (percentage). BPA, Balloon pulmonary angioplasty; dPAP, Diastolic pulmonary arterial pressure; EF, Ejection fraction; LA, Left atrium dimension; LVED, Left ventricular end-diastolic diameter; mPAP, Mean pulmonary arterial pressure; mRAP, Mean right atrial pressure; NT-proBNP, N-terminal pro-brain natriuretic peptide; PAWP, Pulmonary arterial wedge pressure; PVR, Pulmonary vascular resistance; RVED, Right ventricular end-diastolic diameter; 6MWD, 6-min walk distance; S_a_O_2_, Arterial oxygen saturation; sPAP, Systolic pulmonary arterial pressure; S_V_O_2_, Mixed venous oxygen saturation; TRV, Tricuspid regurgitation velocity; VE/VCO_2_ slope, Minute ventilation/ carbon dioxide output slope; VO_2_@Peak, Peak oxygen consumption; WHO FC, World Health Organization functional class. Bold values mean their *P* value < 0.050.
