# Supplementary figures and images for: Diffusing Capacity for Carbon Monoxide Predicts Response to Balloon Pulmonary Angioplasty in Patients With Inoperable Chronic Thromboembolic Pulmonary Hypertension
Source: Front Cardiovasc Med. 2021 Dec 2;8:762267. doi: 10.3389/fcvm.2021.762267 (PMC8674470; doi:10.3389/fcvm.2021.762267)

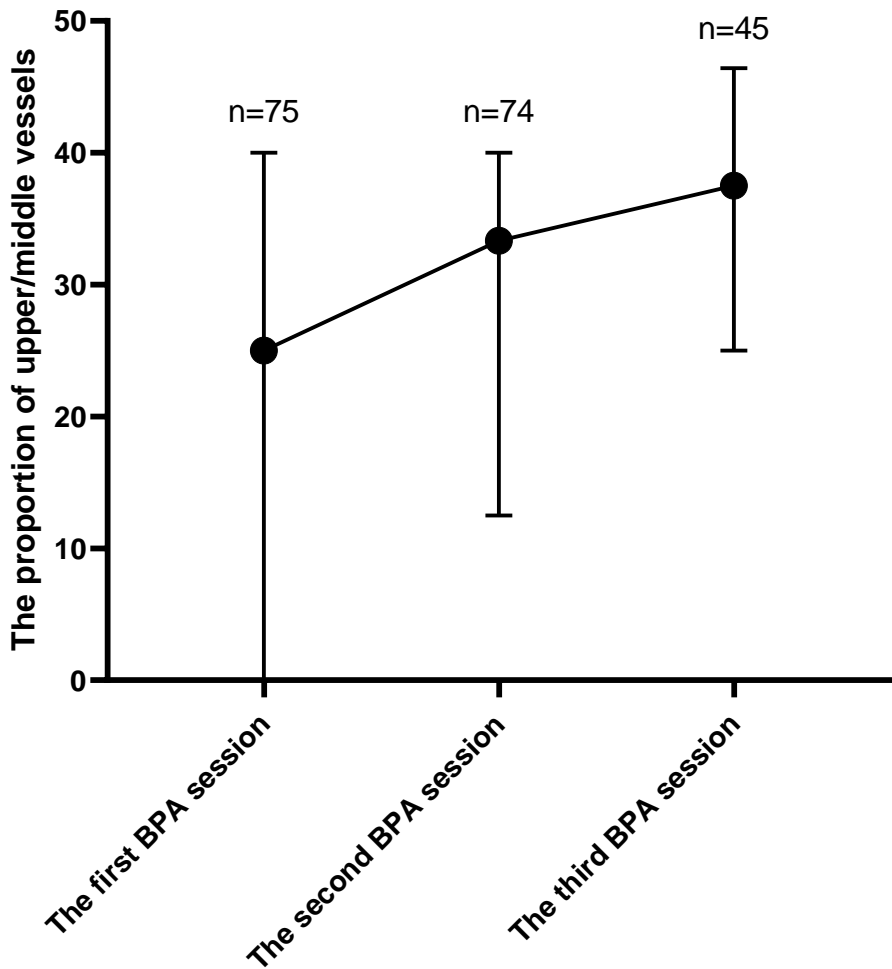

Supplement: Supplementary file 2 [file Image_1.PDF]
